# Acoustic perception and emotion evocation by rock art soundscapes of Altai (Russia)

**DOI:** 10.3389/fpsyg.2023.1188567

**Published:** 2023-09-19

**Authors:** Samantha López-Mochales, Raquel Aparicio-Terrés, Margarita Díaz-Andreu, Carles Escera

**Affiliations:** ^1^Brainlab – Cognitive Neuroscience Research Group, Department of Clinical Psychology and Psychobiology, Universitat de Barcelona (UB), Barcelona, Spain; ^2^Institut de Neurociències, Universitat de Barcelona (UB), Barcelona, Spain; ^3^Departament d’Història i Arqueologia, Universitat de Barcelona (UB), Barcelona, Spain; ^4^Institució Catalana de Recerca i Estudis Avançats (ICREA), Barcelona, Spain; ^5^Institut d’Arqueologia de la Universitat de Barcelona (IUAB), Universitat de Barcelona (UB), Barcelona, Spain; ^6^Institut de Recerca Sant Joan de Déu (IRSJD), Esplugues de Llobregat, Spain

**Keywords:** acoustic perception, emotion, rock art, psychoacoustics, archaeoacoustics

## Abstract

The major goal of psychoarchaeoacoustics is to understand the psychology behind motivations and emotions of past communities when selecting certain acoustic environments to set activities involving the production of paintings and carvings. Within this framework, the present study seeks to explore whether a group of archaeological rock art sites in Altai (Siberia, Russia) are distinguished by particular acoustic imprints that elicit distinct reactions on listeners, in perceptual and emotional terms. Sixty participants were presented with a series of natural sounds convolved with six impulse responses from Altai, three of them recorded in locations in front of rock art panels and three of them in front of similar locations but without any trace of rock art. Participants were interrogated about their subjective perception of the sounds presented, using 10 psychoacoustic and emotional scales. The mixed ANOVA analyses carried out revealed that feelings of “presence,” “closeness,” and “tension” evoked by all sounds were significantly influenced by the location. These effects were attributed to the differences in reverberation between the locations with and without rock art. Despite results are not consistent across all the studied rock art sites, and acknowledging the presence of several limitations, this study highlights the significance of its methodology. It stresses the crucial aspect of incorporating the limitations encountered in shaping future research endeavors.

## 1. Introduction

An emerging avenue of inquiry posits that investigating sound in the past from different perspectives –acoustics, psychoacoustics, ethnomusicology, etc.– may provide crucial information to better understand past societies (Scarre and Lawson, 2006). In particular, archaeology has given rise to a sub-field of research, archaeoacoustics, a novel perspective interested in sound in the past. Some researchers working in archaeoacoustics are focusing their attention on the acoustics of rock art sites in order to answer questions such as how and why past communities chose certain acoustic environments to decorate and most likely to set social and ritual activities related to the production of paintings and carvings in them (Díaz-Andreu and Mattioli, 2015). Sound affects the way people engage with, transform and create environments or places (Blake and Cross, 2015). Environmental acoustics can affect listeners’ emotional reactions and sound perception (Västfjäll, 2012) and, although sound is an ephemeral event, acoustic environments persevere over time (Rainio et al., 2018). Acoustic and perceptual sciences can be applied together to better understand the sensory potential of a soundscape, by reproducing the conditions of the auditory experience endured by ancient communities through experimentation on modern-day participants (Kolar, 2013). When these approaches combine with the experimental methods of psychology, arguably they give rise to an emerging discipline, that of psychoarchaeoacoustics (Valenzuela et al., 2020).

By recording the so-called impulse response or IR, the acoustic signature of a space of archaeological interest can be recreated (Alvarez-Morales et al., 2020; Díaz-Andreu et al., 2022a,b; Alvarez-Morales et al., 2023; Santos da Rosa et al., 2023). The IR method measures sound propagation between an emission point and a receiver device (Farina, 2007), and allows the recreation of an acoustic space in laboratory conditions through the process of auralization (Vorländer, 2008). Auralization consists in convolving a sound with the IR of interest and presenting listeners with the resulting sound, in order to immerse them in the recorded sonic space (Farina and Ayalon, 2003). Hence, the listeners’ individual reactions, subjective interpretations and affective responses to the sound properties of spaces can be measured (Västfjäll et al., 2002; Pätynen and Lokki, 2016) to ultimately extract conclusions about the election process of the most suitable places for the production of rock art, as in the present study. Hence, the aim of the present research is to investigate from a psychoarchaeoacoustical point of view (Valenzuela et al., 2020), the acoustic environment of a particular rock art area in the Republic of Altai (Russia).

There is a tight relationship between rock art, sound and the landscape with links to the beliefs of the people who produced and experienced the art. These connections have been seen in many cultures around the globe (Díaz-Andreu and Mattioli, 2017; Díaz-Andreu et al., 2021). In Altai, soundscapes and acoustic phenomena had a significant cultural role, at least in historical times, and presumably in prehistoric times. The ethnographic accounts of Altai describe how natural and supernatural entities are related through sound in an ordinary basis, which shapes music esthetics: songs and singing styles imitate natural sounds and, at the same time, nature interacts and imitates human sound with phenomena such as echoes (Díaz-Andreu et al., 2022a,b). Although no explicit mention of rock art is made in the ethnographic sources, in some areas of Altai it is still considered wrong to modify the environments where carved stones are found. This supports the idea that the marking of the stones was part of the described spiritual and ritualistic imaginary, involving sound production and interaction with the acoustic environment (Díaz-Andreu et al., 2022a,b). The rock art of this area is characterized by carvings made in a broad period ranging from, probably, the Neolithic to the Turkic era and even later. The major period of production was from 3,000 BCE to ca 500 CE. All the sites mentioned in this article had rock art motifs from all the different periods, indicating that they were repeatedly visited by many generations since prehistory (Miklashevich and Bove, 2010; Díaz-Andreu et al., 2022a,b). This is the case of Kalbak-Tash I, Kalbak-Tash II, Grand Yaloman and Torgun I-5, The only exception is the panel of Urkosh IVb with only a few carvings of undiagnostic chronology. Still, it is important to note that it is found in a more extensive are with many other rock art panels distinguished, as the previous sites, by having a large quantity of motifs made during a long period of time.

As discussed, some acoustic phenomena are linked to the consideration of a place as a specific location in which social and ritual activities may have taken place (Díaz-Andreu and Mattioli, 2017; Díaz-Andreu et al., 2021). They were not dwelling places, but locales where people undertook activities that had a special sacred or social character and marked the space with paintings or carvings. In our case study, excavations have shown that at the time the rock art was produced there were episodic periods in which fires were lit, pots were used, and sacrifices were performed close to the decorated panels (Surazakov, 1996; Borodovskiĭ et al., 2016). In the present study, the point of departure is the proposal that by exposing listeners to natural sounds in presence of the acoustics from the rock art sites, and studying its effects –in terms of auditory subjective experience and emotion-, we might better understand the auditory experience lived by the communities who took part on the production of rock art and engaged with it later on. The objective of the present study was hence to observe which specific psychoacoustic and emotional dimensions were experienced differently in the presence of the soundscape of a spot close to a rock art panel, compared to a spot located away from it.

For our experiment, we selected a set of 17 natural sounds present in the natural landscapes of Siberia (including sounds from autochthonous animals and weather phenomena). We decided to use natural sounds to avoid cultural biases that could be caused by the use of music (Zentner et al., 2008; Hagman, 2010; Carew and Ramaswami, 2020). In particular, the music from the region of Altai could be esthetically unpleasant for modern-day listeners not familiar with the cultural tradition in Altai and neighboring areas. Sounds were convolved with six different IRs from Altai, three of them recorded in front of wall marked with carvings on the rocks, and three from wall of similar physical characteristics but not marked with rock art carvings. Experiment participants were presented with these sounds and were interrogated about their feelings and perception using 10 psychoacoustical and emotional descriptors. The sounds were presented in a spatial audio format –3rd order Ambisonics– to recreate the acoustic environments more accurately and to enhance stronger emotional reactions (Västfjäll, 2003).

Not only sounds (Bradley and Lang, 2000) play a role in emotion evocation, but also other-than-content features of sound –for example, physical attributes of sound propagation in space (Västfjäll, 2003). Therefore, in our experiment we expected to observe significant variations in all the psychoacoustical and emotional dimensions studied when our participants listened to the sounds convolved with the different IRs. Moreover, we expected to find disparities in these dimensions when listening to the sounds convolved with the IRs recorded in front of the rock art panels (from now on, *art+*) compared to the ones recorded away from the rock art panels (from now on, *art−*). Finding contrasting perceptions between the *art+* and *art−* sounds, and commonalities within each group, would be a first step toward considering that acoustics played some role in the selection of the most suitable places for rock art production in the study area in Altai and its related activities. Furthermore, it would reveal which dimensions of perception and emotion we should keep exploring to better understand the experience endured by individuals who took part on the production of rock art carvings.

Considering the numerous limitations inherent in the present study, it becomes imperative to acknowledge and address these constraints from the outset. Firstly, the IRs used were recorded long after rock art creation, potentially impacting landscape accuracy. Geomorphological and climatic changes might have occurred in the course of hundreds of years, as well as changes in the vegetation of the area, influencing sound absorption and, consequently, acoustical properties. Secondly, the accuracy of soundscape reconstructions is uncertain due to unavailable anechoic or quasi-anechoic recordings from natural sources for convolutions. Lastly, the empirical approach to study emotions in ancient societies raises concerns about cultural differences. Despite it presents limitations, and its interpretations should be cautious, this study provides valuable insights into acoustic considerations in the process of rock art production by past societies. However, interpretations should be cautious, recognizing the constraints of the present perspective.

## 2. Methods

### 2.1. Participants

Sixty healthy human volunteers −44 women and 16 men of ages between 18 and 31 years old– participated on this experiment. The recruitment was carried out via students and research staff mail lists, blogs and social media. The exclusion criteria for participants included hearing impairments, psychiatric or neurological illnesses, ages below 18 or above 35 years and consumption of drugs or pharmaceuticals acting on the central nervous system. The study was approved by the Bioethics Committee of the University of Barcelona. All methods were performed in accordance with the relevant guidelines and regulations. All participants gave written informed consent in compliance with the Code of Ethics of the World Medical Association (Declaration of Helsinki).

### 2.2. Stimuli

#### 2.2.1. Sounds

The stimulus set was composed of 17 natural sounds of 20 s of duration, including animals (a bear, a crane, a plover, a cricket, an eagle, an ibex, a leopard, a lynx, a sheep and a pack of gray wolves) and other natural phenomena (a bonfire, the rain, a river, a snowstorm, a thunderstorm, a waterfall and the wind). The sounds were extracted from several sources; details are provided in Supplementary Table S1.

Five additional sounds (a dunnock, a baboon, a river, an impala and a forest atmosphere) were used in the first five trials of each block, but the data obtained from these trials were not included in the analysis. These five trials were used to allow participants to get immersed in the two different acoustic conditions (*art+* and *art−*), and to ensure that the task to be carried out was clearly understood (Bradley et al., 1999; Witew et al., 2005; Vigeant and Celmer, 2010; Marquis-Favre et al., 2019). These additional sounds were extracted from the BBC sound effects repository; details are provided in Supplementary Table S2. The amplitude of sounds was normalized using Audacity^1^ version 2.3.2.

#### 2.2.2. Impulse responses

**Figure 1:**
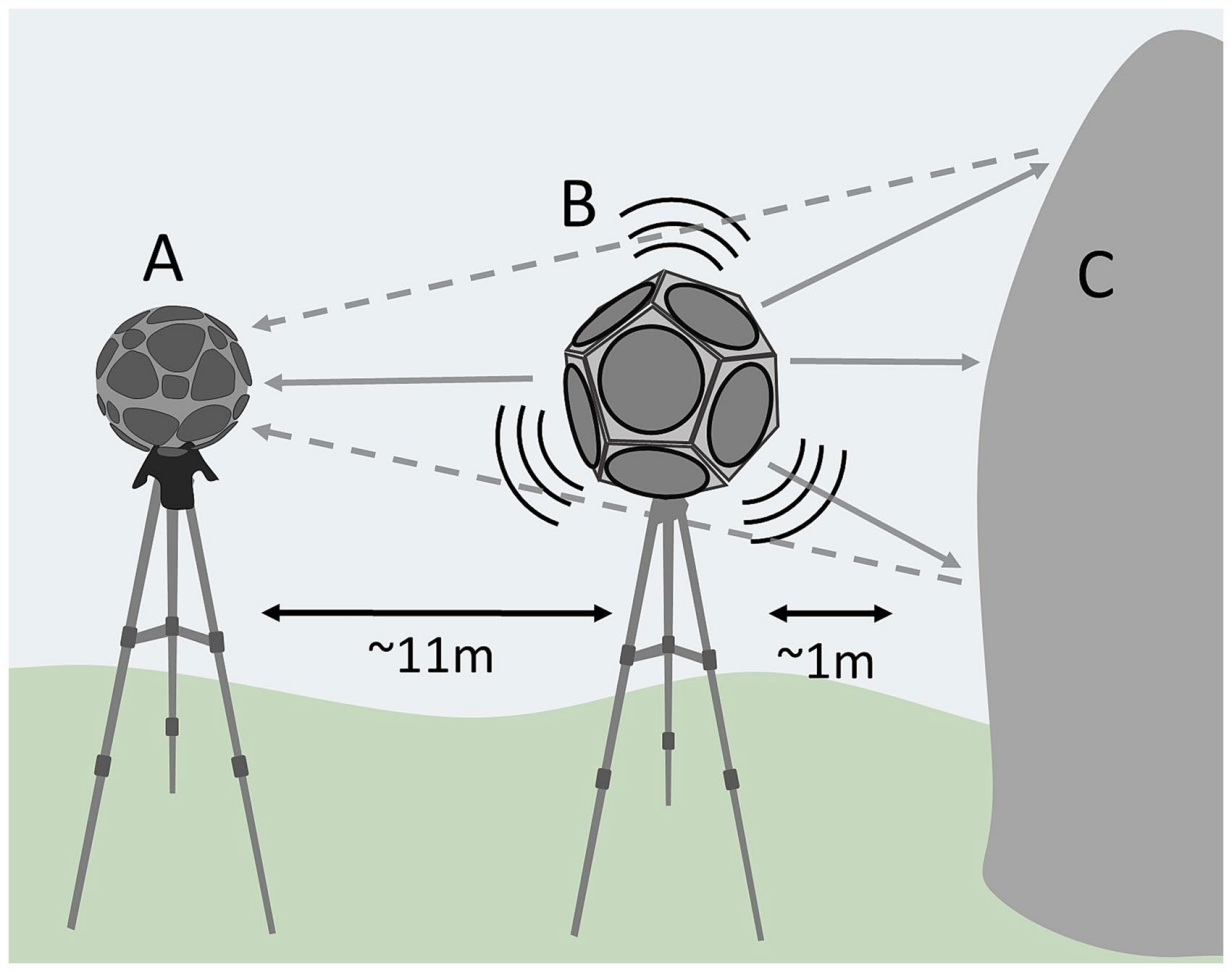
Schema of the recording position of the Ambisonics microphone **(A)** and the loudspeaker **(B)** with respect to the rock faces **(C)**.

Each sound of the stimulus set was convolved with six different IRs recorded in different natural landscapes across the Republic of Altai. All the IRs were recorded and processed using the MIMO IR technique (Farina and Chiesi, 2016; Farina, 2020). The position of the source and the microphone with respect to the rock faces was constant; the microphone was placed in front of the rock, at least 1 m away from it, and the loudspeaker was placed 10–12 m away from the microphone, aligned with it, and perpendicular to the direction of the panel (Figure 1). Three of the IRs were recorded in front of panels with rock art motifs (*art+*), and the other three were recorded in front of rock panels without any presence of rock art, at least 30 meters away from any wall with carvings on it (*art−*). The objective of this methodological approach was to isolate the effects of the critical variable *presence of rock art*, which would allow attribution of the eventual effects to the presence of the carvings. The six resulting versions of the stimulus set were grouped in three bundles (one, two and three), each including one version convolved with an IR from an *art+* recording position -in front of a rock art panel- and an *art−* recording position -convolved with an IR from a recording position in front of a rock face away from the rock art panels.

**Figure 2:**
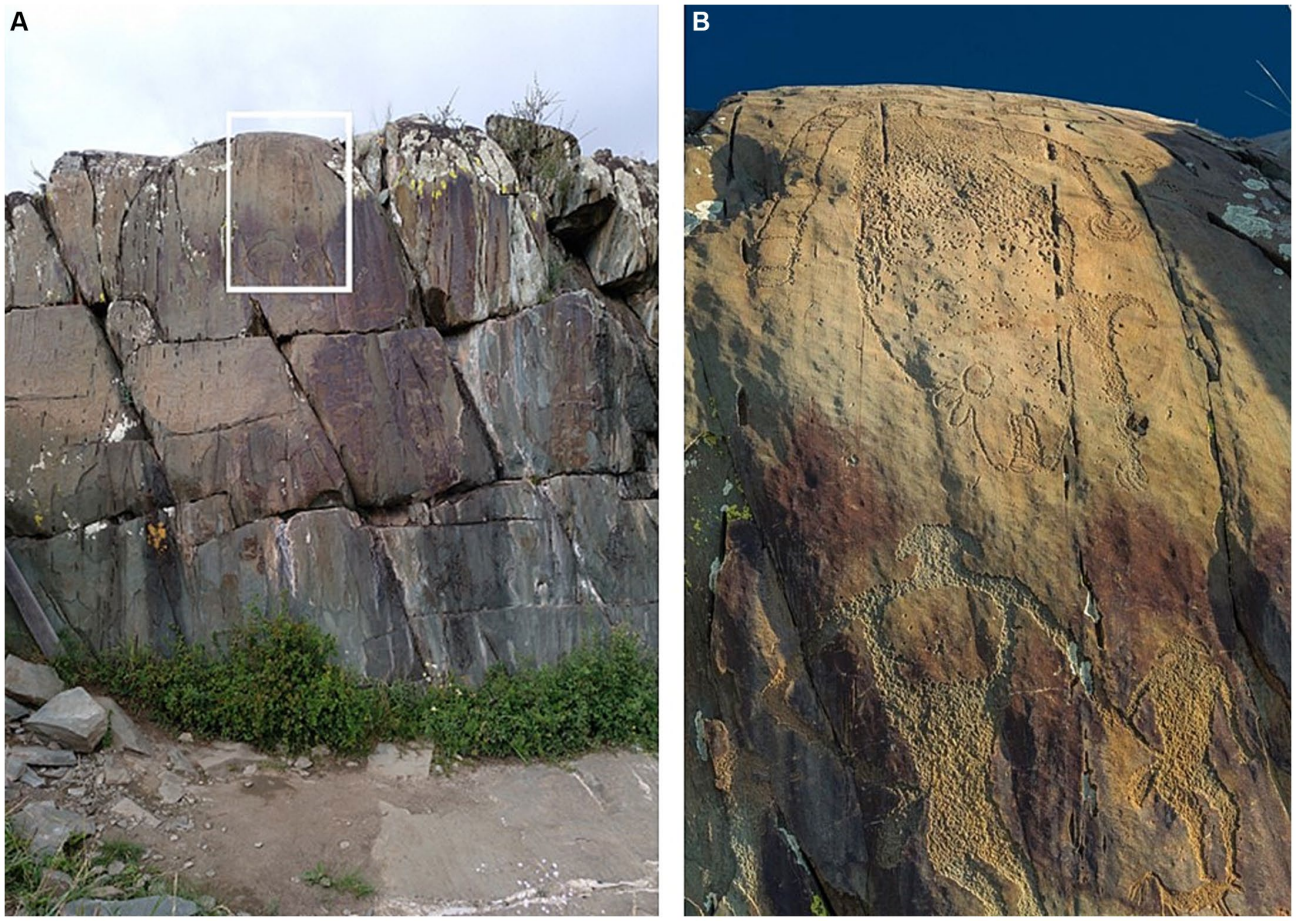
Rock art with the “hanging monster”, in Kalbak-Tash I. The impulse response recorded in front of this rock art panel was employed in the *art*+ version of the first bundle of IRs. **(A)** General view with a rectangle where the view provided on the photograph **(B)** is located. Photo **(A)** by Margarita Díaz-Andreu. Photo **(B)** (detail of panel) by Andrej Rozwadowski.

The first bundle included a version of the stimulus set (*art+* 1) convolved with an IR recorded in front of one of the rock art panels at Kalbak-Tash I (Figure 2, E 86° 49′ 10″, N 50° 24′ 05″), in the area of the Lower Chuya river. Kalbak-Tash I is a rocky flattened hill, located in the lower part of the Chuya River valley, close to the confluence with the Katun river (Díaz-Andreu et al., 2022a). The corresponding *art−* version of the stimulus set (*art−* 1) was convolved with an IR recorded in the area of Urkosh (Díaz-Andreu et al., 2022a, 2022b) called “rock face 2,” in the coordinates E 086° 33′ 36″, N 50° 32′ 50″ and not that far from the rock art site of Grand Yaloman (Díaz-Andreu et al., 2022a), situated in E 86° 34′ 09″, N 50° 33′ 01″.

**Figure 3:**
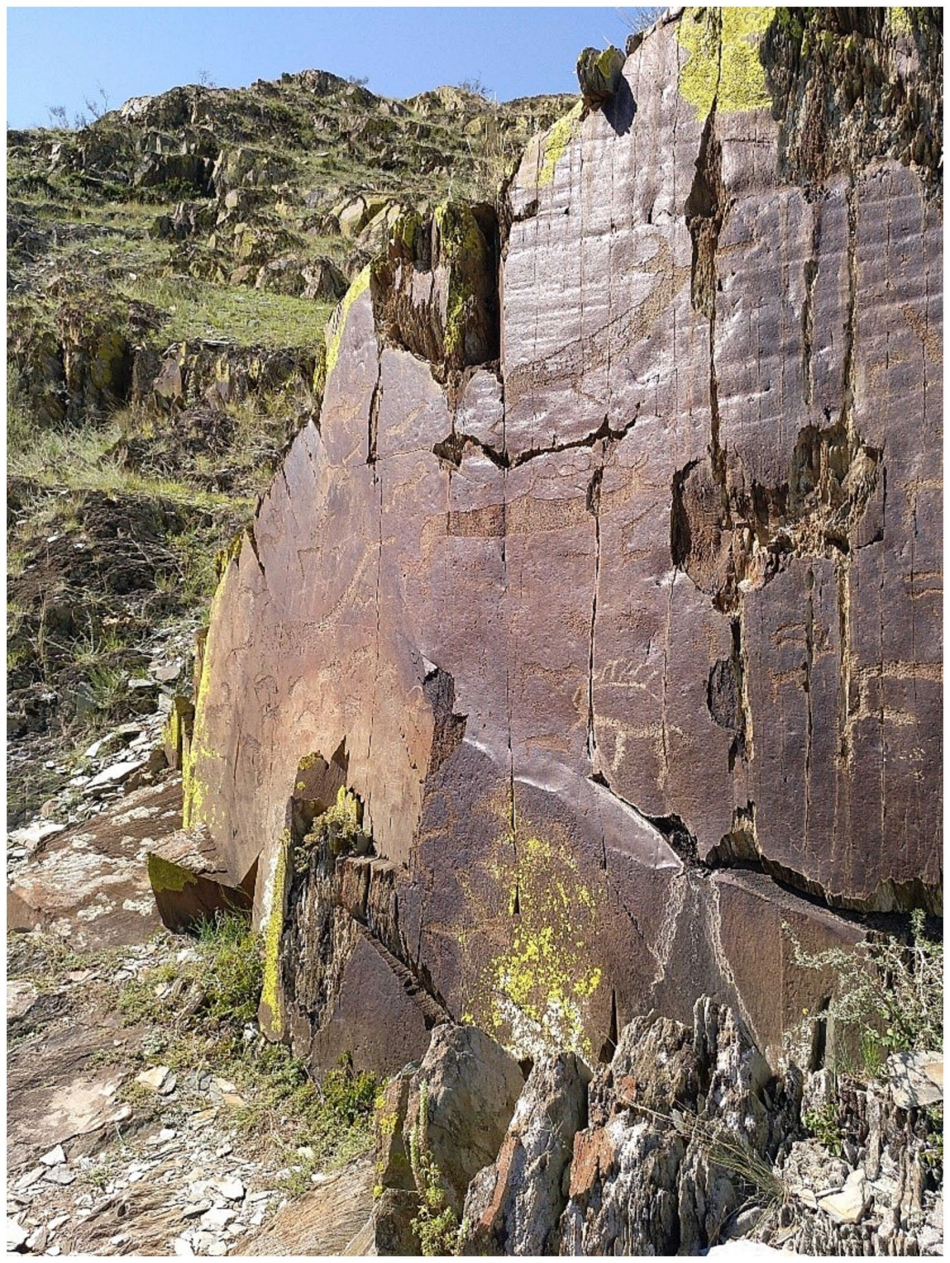
Rock art panel in Kalbak-Tash II, panel 2. The impulse response recorded in front of this rock art panel was employed in the *art*+ version of the second bundle of IRs. Photo by Margarita Díaz-Andreu.

The second bundle included a version of the stimulus set (*art+* 2) convolved with another IR from the area of Kalbak-Tash II (in the Lower Chuya River valley; Díaz-Andreu et al., 2022a), recorded in front of one of the rock art panels of Kalbak-Tash II, panel 2 (Figure 3, E 86° 41′ 20″, N 50° 24′ 13″). The corresponding *art−* version of the stimulus set (*art−* 2) was convolved with an IR recorded in the area of Urkosh, next to the Katun River, in front of a small wall next to a rock panel without rock art, named “rock face 1” (coordinates E 86° 34′ 15″, N 50° 32′ 12″).

**Figure 4:**
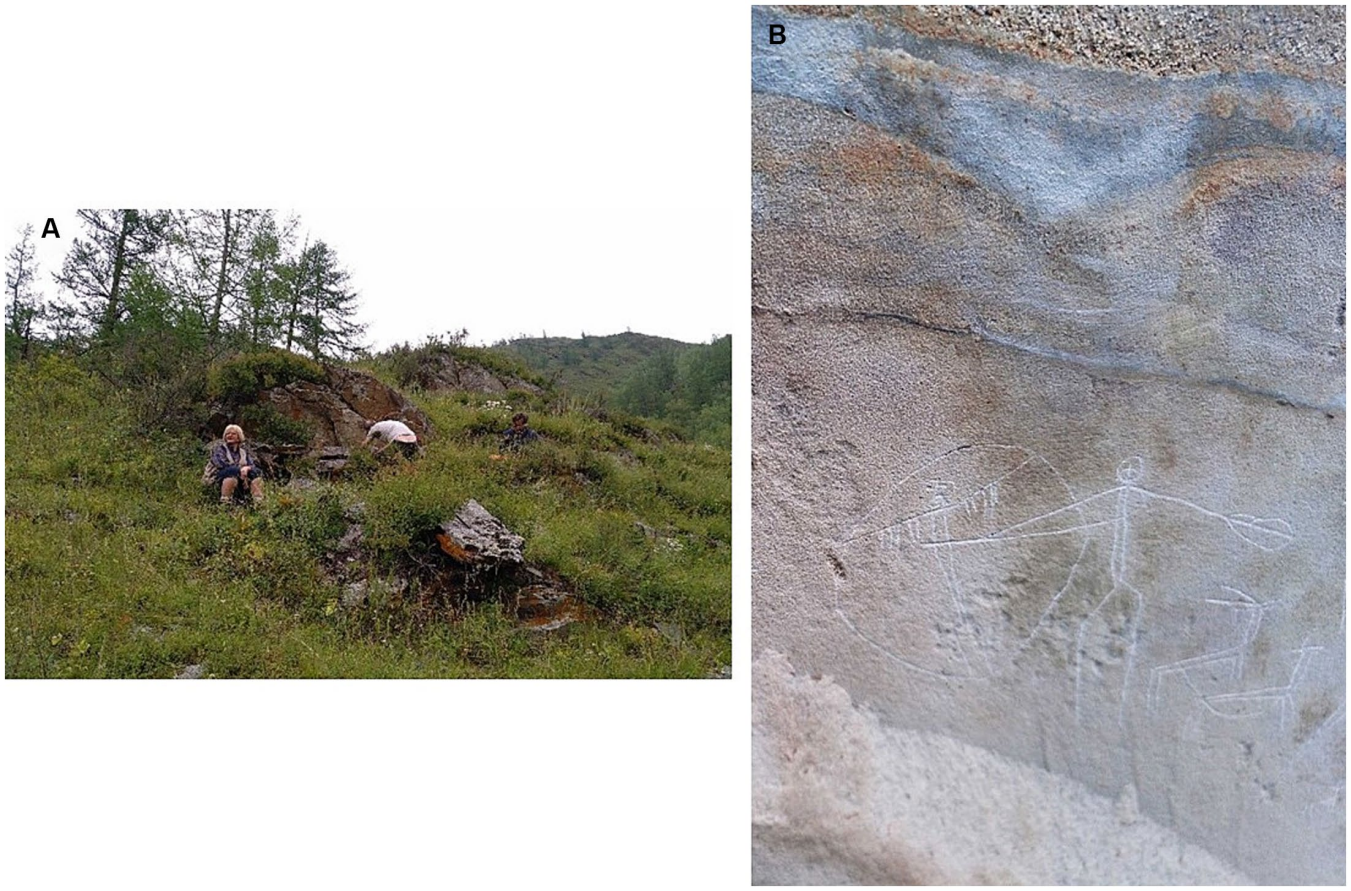
Rock-art panel of Torgun I5, in the Karakol valley. The impulse response recorded in front of this rock art panel was employed in the *art*+ version of the third bundle of IRs. **(A)** General view; **(B)** detail of panel with the representation of a shaman with a drum. Photos by Margarita Díaz-Andreu.

The third bundle included a version of the stimulus set (*art+* 3) convolved with an IR recorded in the lower part of the Karakol River valley, before the junction with the river Ursul, in the area of Torgun –an area composed by several rock art panels located south of the locality of Bichiktu-Boom. The panel chosen was Torgun I5 (Figure 4, E 85° 55′ 18″, N 50° 46′ 13″). The corresponding *art−* version of the stimulus set (*art−* 3) was convolved with another IR from the area of Urkosh (Díaz-Andreu et al., 2022a), close to the rock art panel of Urkosh IVb, but in front of a wall several meters away from the rock art (E 86° 33′ 35″, N 50° 32′ 51″).

**Figure 5:**
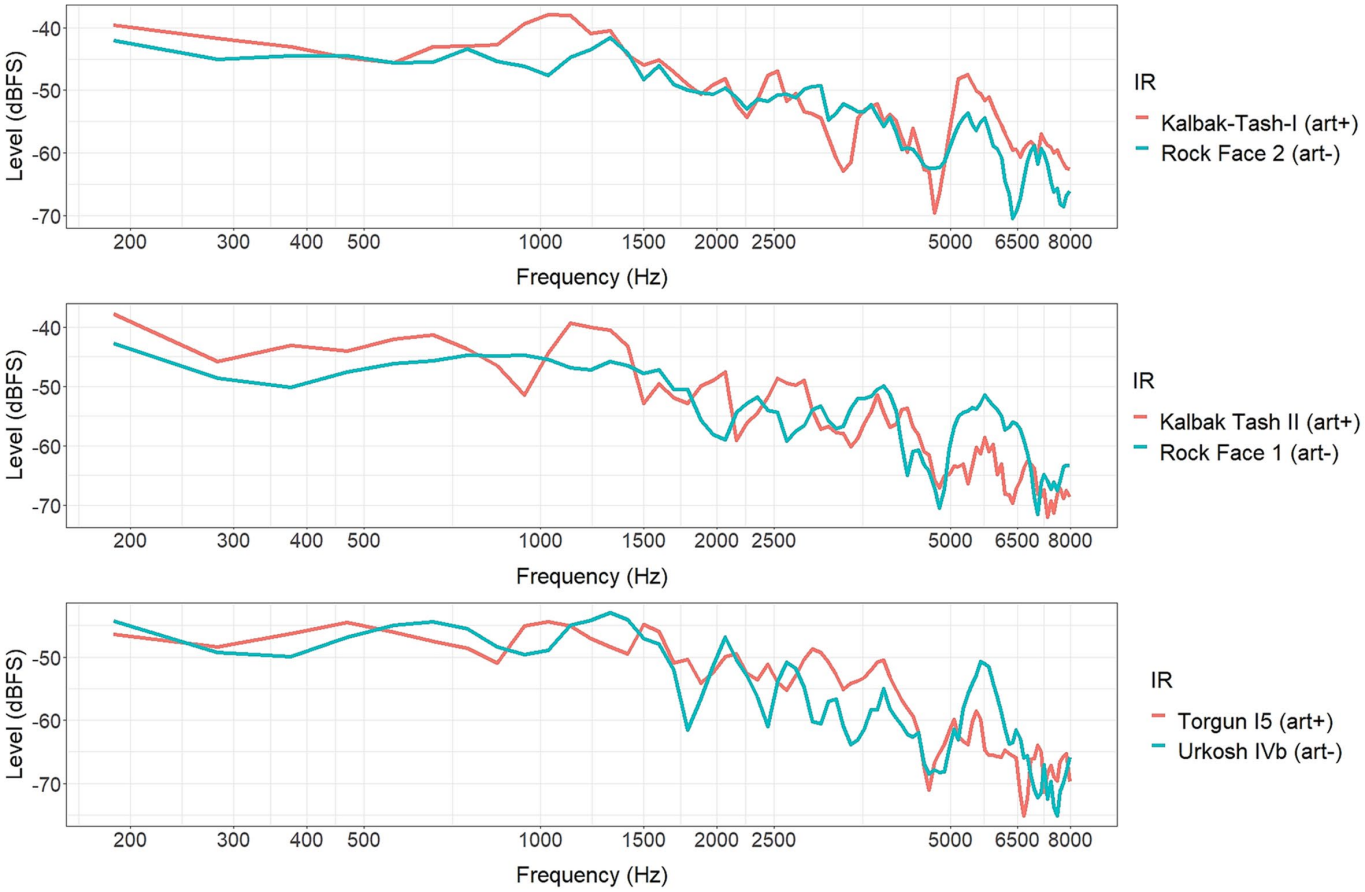
Spectral analysis of each one of the six impulse responses, presented in pairs of *art*+ and *art*− corresponding to each bundle.

Participants were randomly assigned to three groups of 20, and each group was presented with one of the three bundles of stimuli. From the six described IRs, several acoustic parameters [included in ISO 3382-1 standards, Asociación Española de Normalización y Certificación (AENOR), 2010] were retrieved. These parameters were calculated using the ARTA^2^ acoustic software, version 1.9.4.1. A selection of the parameters that show some differences between the *art+* and *art−* IRs is presented in Table 1. These are speech clarity (C50, measured in decibels), music clarity (C80, measured in decibels) and reverberation time (EDT, T20 and T30, measured in seconds). Figure 5 shows the spectral analysis of the six IRs, obtained using Audacity version 2.3.2.

**Table 1:** Values for the acoustic parameters: speech clarity (C50, dB), music clarity (C80, dB), and reverberation time (EDT, T20, and T30, seconds), calculated from the six impulse responses employed in the study.

Group	Art	Site	C50	C80	EDT	T20	T30
G1	art+	Kalbak Tash I	29.108	38.936	0.044	0.118	0.142
	art−	Urkosh wall 2	19.311	21.336	0.048	0.596	0.549
G2	art+	Kalbak Tash II	23.316	27.809	0.081	0.166	0.311
	art−	Urkosh wall 1	12.624	17.616	0.109	0.423	0.342
G3	art+	Torgun I5	27.368	35.084	0.045	0.137	0.171
	art−	Urkosh Ivb	16.474	29.039	0.088	0.228	0.222

Average of the octave bands from 125 to 4,000 Hz.

#### 2.2.3. Convolution

The convolution of the stimuli with the six impulse responses selected was performed using the software Bidule,^3^ by Plogue Art et Technologie and the X-MCFX plugin, version 0.5.11.^4^ The format of the original audio files was mono WAV, and the format of the IRs was 3rd order Ambisonics (Schacher and Kocher, 2006). After the convolutions, it was verified that any of the resulting sounds produced any clipping. The amplitude of the sounds was not normalized after the convolutions; this way, we preserved any amplifications of particular frequency bands that could be generated from the convolution and that could have effects on the affective responses of listeners.

### 2.3. Rating scales

In order to identify the most suitable emotional and psychoacoustical descriptors for participants to rate the sensations experienced during the listening, a pilot experiment was performed. A pre-selection of items was first made, with 14 items from several validated scales, such as the Circumplex Model of Affect or the Geneva Emotional Musical Scale, that have been employed in many previous behavioral studies with auditory stimuli (Russell, 1980; Watson and Clark, 1988; Zentner et al., 2008; Barron, 2009; Trost et al., 2012; López-Mochales et al., 2022). These items were: *presence*, *spaciousness*, *envelopment*, *deepness*, *closeness*, *darkness*, *fright*, *alert*, *tension*, *peace*, *power*, *transcendence*, *calm*, and *pleasure*. Other items from the mentioned scales, such as *joy*, *nostalgia* or *wonder*, were disregarded, because were considered irrelevant for describing sounds without any cultural meaning. Fourteen individuals participated in the pilot experiment (eight women and six men) of ages between 22 and 30 years. They listened to five excerpts of 20 s duration of natural sounds, not convolved with any impulse response, including an insect, a mammal, a bird, a river and the ambient sound of a storm. These sounds were selected from the BBC sound effects repository, and the details are provided in Supplementary Table S3. Pilot participants had to rate how they felt during the listening of each one of the sounds using the 14 pre-selected psychoacoustical and emotional descriptors in scales from 1 to 10, 1 being “not at all” and 10 being “very much.” At the end, the pilot participants had to indicate which three descriptors had been the most “clearly ratables” for each one of the sounds, to determine the most relevant psychoacoustical and emotional dimensions to interrogate in our actual experiment in order to study the reactions of participants to this type of natural sounds.

From the results of the pilot study, we selected the descriptors that pilot participants classified as the top three most “clearly ratable.” We came out with the following final list of 10 descriptors: *presence*, *spaciousness*, *envelopment*, *deepness*, *closeness*, *alert*, *tension*, *peace*, *calm*, and *pleasure*. The other four descriptors, *darkness*, *fright*, *power*, and *transcendence*, were disregarded, because did not appear in any of the pilot participants’ top three.

### 2.4. Procedure

**Figure 6:**
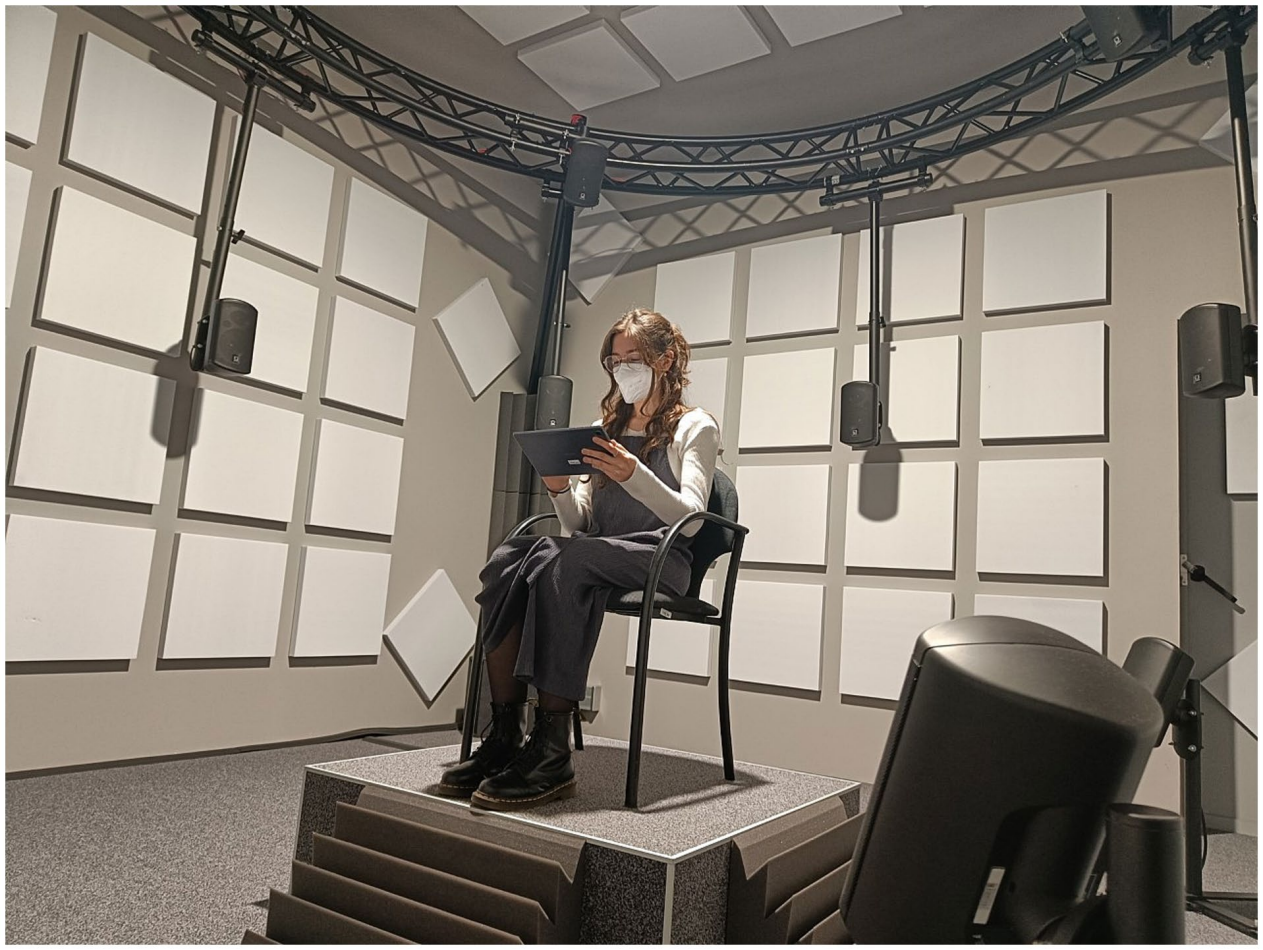
Listening room of the Immersive Psychoacoustics Laboratory of the Faculty of Psychology of the University of Barcelona.

The experiment was carried out in the *Immersive Psychoacoustics Laboratory* (immpaLAB, Figure 6), located in the Faculty of Psychology of the University of Barcelona (Spain). This facility, consists of a control room and a soundproofed chamber. The latter has a 16 loudspeakers array, disposed in a 3D sphere and equidistant from the participant’s head, that allow rendering sound in third order Ambisonics format. Experiment participants were asked to sit in the center of the soundproofed chamber.

Before starting the experimental session, participants were requested to fill in a series of questionnaires. Since in Western culture, individuals tend to associate long reverberation times with sacred spaces (Baumann and Niederstätter, 2008; Ghaffari and Mofidi, 2014), and reverberation is one of the differential features of some of the stimuli employed in the present study, the sense of religiosity and spirituality of participants was accounted by means of a questionnaire [the Centrality of Religiosity Scale (Huber and Huber, 2012)]. As participants’ level of anxiety could interfere with some of their responses –especially to those sounds and descriptors proven to cause such feeling (Toprac and Abdel-Meguid, 2010)-, this was taken into account, by means of the State–Trait Anxiety Inventory (STAI, Spielberger et al., 1983). For each individual we calculated an index of religiosity (ranging 0 to 28) and the two scores derived from the STAI: a *state* score, s-STAI, to assess anxiety associated to specific situations, and a *trait* score, t-STAI, to assess the presence of the personality trait that reflects a tendency to suffer state anxiety (Spielberger et al., 1983).

After responding to all questionnaires, participants read the instructions of the experiment, describing the task to be performed, and a definition of each one of the psychoacoustical and emotional labels to be rated. Each participant was assigned to one of the three groups and, therefore, presented with one of the three bundles of stimuli described previously. Stimuli were presented in random order, first in one of the two conditions (*art+* or *art−*) and then with the stimuli in the same order in the other condition. Half participants listened to the sounds presented in the *art+* condition first and in the *art−* condition second, and the other half, the other way around. Participants listened to a total of 44 sounds of 20 s length (a set of 17 sounds, plus five additional sounds played at the beginning, all presented in two conditions). The volume was adjusted at a comfortable level by experimenters, and sounds were presented at the same level to all participants. After listening to each sound, participants had to rate the 10 descriptors mentioned above, according to how they perceived the sound and how it made them feel. Each descriptor was rated from 0 (meaning “not at all”) to 10 (meaning “very much”).

**Figure 7:**
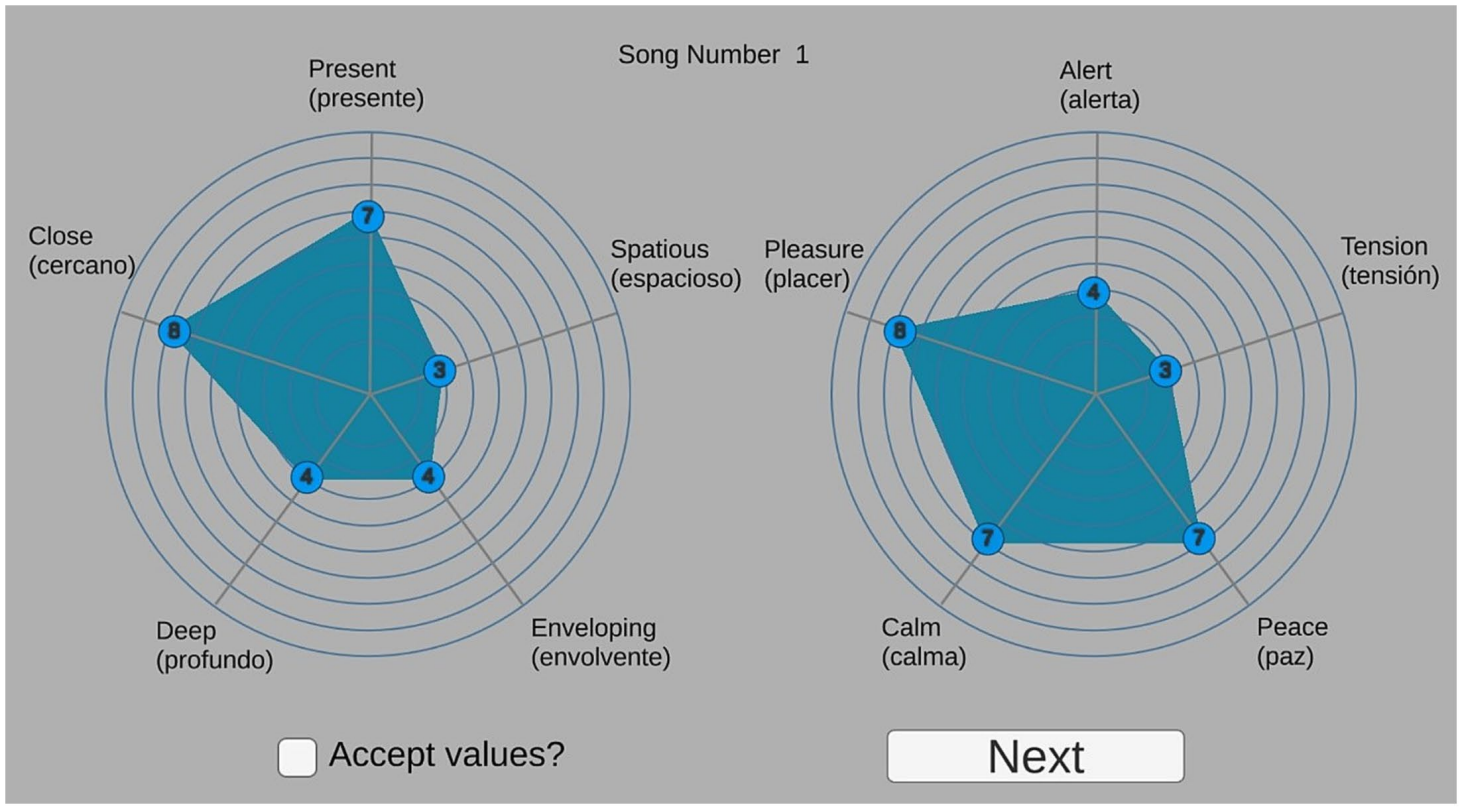
Tablet screen with customable spider charts for rating the descriptors of the experiment.

The experiment was carried out using an electronic tablet. The ratings were obtained using two customable spider charts, one for the psychoacoustical labels and another for the emotional labels, with five radii each (Figure 7). Each radius represented one of the descriptors, with the corresponding label at the end. Participants had to drag and drop a pointer to a specific position along the radius, indicating the punctuation they wanted to assign to each descriptor, from 1 (dragging and dropping the pointer to the center of the circle) to 10 (dragging and dropping the pointer to the end of the radius). This method for rating behavioral scales was chosen over a text version so that the process of selecting/confirming options with multiple assessments were speeded and undertaken in a short time. A total of 44 screens, one for each sound, were presented in the tablet, and the results were automatically saved in a CSV file. The experiment had an approximate duration of 75–90 min.

### 2.5. Statistical analysis

In order to consider the scores *religiosity*, *sSTAI* and *tSTAI*, derived from the questionnaires presented to participants, as covariates in the models, each score’s effect on the response variables was studied using linear regression. Observing the outcome of the analyses, no effects of the scores were found on the ratings of the descriptors (see Supplementary Table S4). Therefore, the scores were not included as covariates in the models.

A total of 10 mixed ANOVA analyses were carried out, one for each psychoacoustical and emotional descriptor. This approach considered the analysis of the ratings for each one of the 10 descriptors as responding to a different research question separately, addressing participants’ perception of auralizations from *art+* spots as more or less *present*, *spatious*, *enveloping*, *deep*, *close*, and evoking more or less *alert*, *tension*, *peace*, *calm*, and *pleasure*. Although the scorings of the descriptors were ordinal, they were considered as intervals as recommended by Carifio and Perla, 2007. Even though some of the data groups did not distribute normally, we opted to use parametric methods, which are deemed robust for the analysis of data from scales with ordinal scorings (Norman, 2010), and have been used in previous similar studies (Miu and Balteş, 2012; Pearce and Halpern, 2015; Hanser et al., 2016). Other reasons to disregard the normality assumption are the large sample size and that the number of observations was constant across the groups (Feir-Walsh and Toothaker, 1974; Blanca et al., 2017). On each one of these 10 models, the response variable was the *descriptor* rating, and two within-subject factors -*sound* (with 17 levels, corresponding to each of the sounds that formed the stimulus set), and *art* (with two levels, *art+* and *art−*)- and one between-subject factor -*group* (with three levels)- were included.

*Post-hoc* comparisons, using Wilcoxon Signed-Ranks tests, were performed for an in-depth study of the factors’ interactions. All the *p*-values were corrected for multiple testing using the Benjamini-Hochberg (FDR) method. In all cases, the significance level considered was α = 0.05. All the analyses were carried out using the software R (version 3.6.2) and the *ez* package (Lawrence, 2016).

Since some of the significant results obtained in the *post-hoc* comparisons concerned the ratings attributed to specific sounds from the stimulus set, the frequency spectrum of these sounds after their convolution was analyzed, using Audacity1 version 2.3.2, to help in the interpretation of the results.

## 3. Results

### 3.1. Behavioral experiment

After carrying out the mixed ANOVA analyses on our experimental data, including the within-subject factors sound and art, and the between-subject factor group, all the descriptors’ ratings were influenced by the single factor sound. However, for the interests of the present study, the implications of the isolated effect of the factor *sound* were not further developed. Instead, the focus was put on the interactions between all the factors of the experimental design, including *sound* as well as *art* and *group*.

A significant effect of the factor of interest (*art*) was observed over the ratings of the descriptors *present* [*F*(1,57) = 4.845, *p* = 0.0454], *close* [*F*(1,57) = 15.872, *p* = 0.0010], *alert* [*F*(1,57) = 8.131, *p* = 0.0101], *tension* [*F*(1,57) = 27.876, *p* = 2.1·10^−5^], *peace* [*F*(1,57) = 13.026, *p* = 0.0020], *calm* [*F*(1,57) = 12.550, *p* = 0.0020] and *pleasure* [*F*(1,57) = 10.503, *p* = 0.0040]. However, the low values of η2 indicate that this factor, isolated, does not explain much of the variability in the response variable. Therefore, the focus was put on studying the effect of factors’ interactions by carrying out *post-hoc* tests. The summary of all mixed ANOVA analyses is presented in Supplementary Table S5.

A significant influence of the interaction of the factors *art* and *group* was found in the ratings of the descriptors *present* [*F*(2,57) = 6.415, *p* = 0.0154], *close* [*F*(2,57) = 11.565, *p* = 0.0006] and *tension* [*F*(2,57) = 5.653, *p* = 0.0192]. *Post-hoc* analyses were performed for the ratings of each one of these descriptors, using Wilcoxon Signed-Ranks tests. We analyzed the effect of the factor art on each group separately.

**Figure 8:**
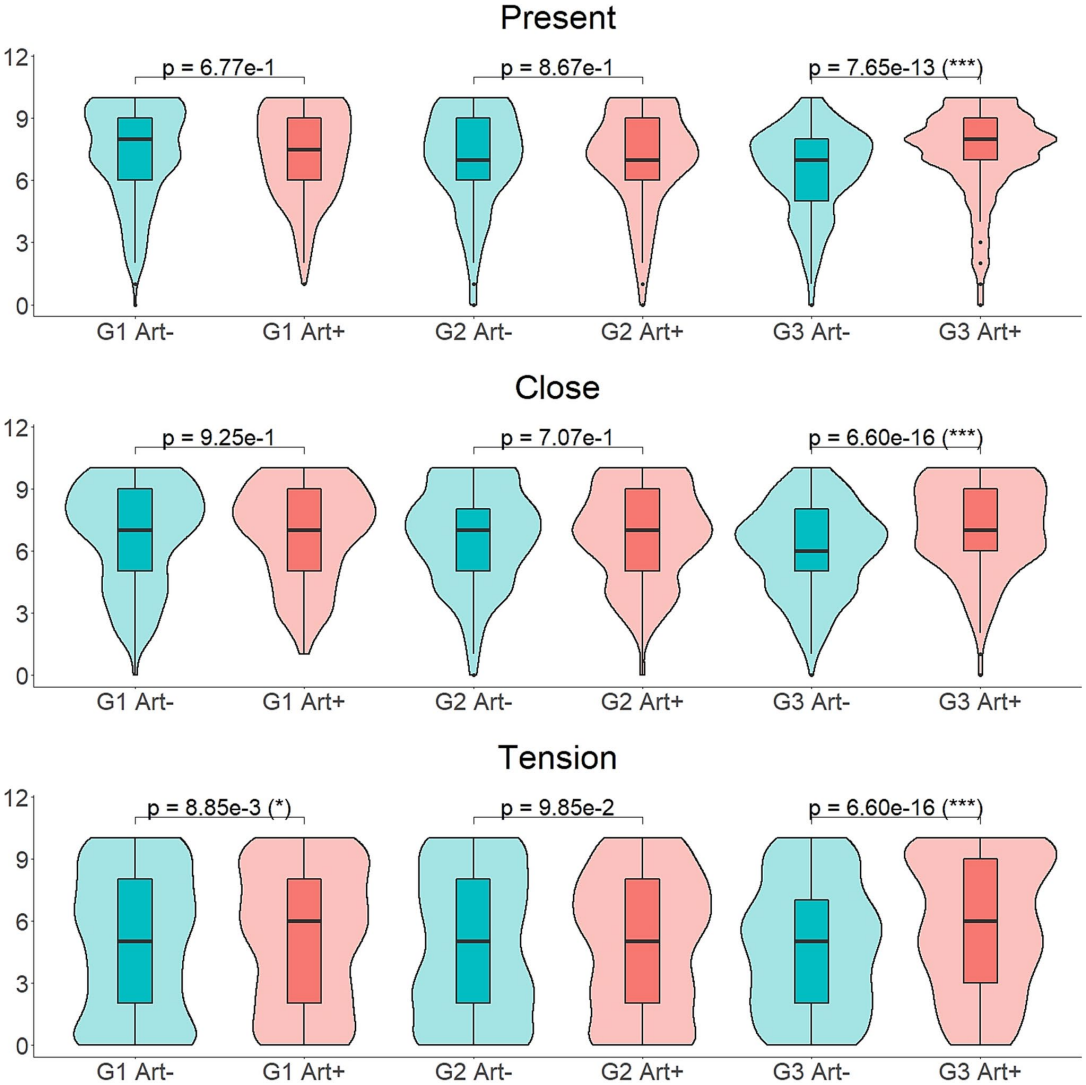
*Post-hoc* comparisons of the ratings of the descriptors *present*, *close* and *tension* in the different experimental groups, using Wilcoxon Signed-Ranks tests to study the differences between the *art*+ and *art*− conditions. The *p*-values displayed have been corrected for multiple testing.

For the descriptors *present* and *close*, significant differences were found between the values attributed to the *art+* and *art−* versions of the stimuli, in the third experimental group solely. For the descriptor *tension*, significant differences were found between the values attributed to the *art+* and *art−* versions of the stimuli, in the first and third experimental groups. Details of the analyses are presented in Supplementary Table S6. Participants from the third experimental group rated the sounds convolved with the impulse response recorded in front of the rock art panel of Torgun I5 (*art+*) as more *present* (*M* = 7.338, SD = 2.118), *closer* (*M* = 7.182, SD = 2.124), as well as evoking more *tension* (*M* = 5.747, SD = 3.174) in comparison to the *art−*, in which the sounds were rated as less *present* (*M* = 6.511, SD = 2.065), less *close* (*M* = 6.171, SD = 2.193) and evoking less *tension* (*M* = 4.774, SD = 3.047). Participants from the first experimental group also rated the sound convolved with the *art+* IR recorded in front of the rock art panel of Kalbak-Tash I as evoking more *tension* (*M* = 5.279, SD = 3.265) compared to the *art*− version of the sounds (*M* = 4.915, SD = 3.388; Figure 8).

**Figure 9:**
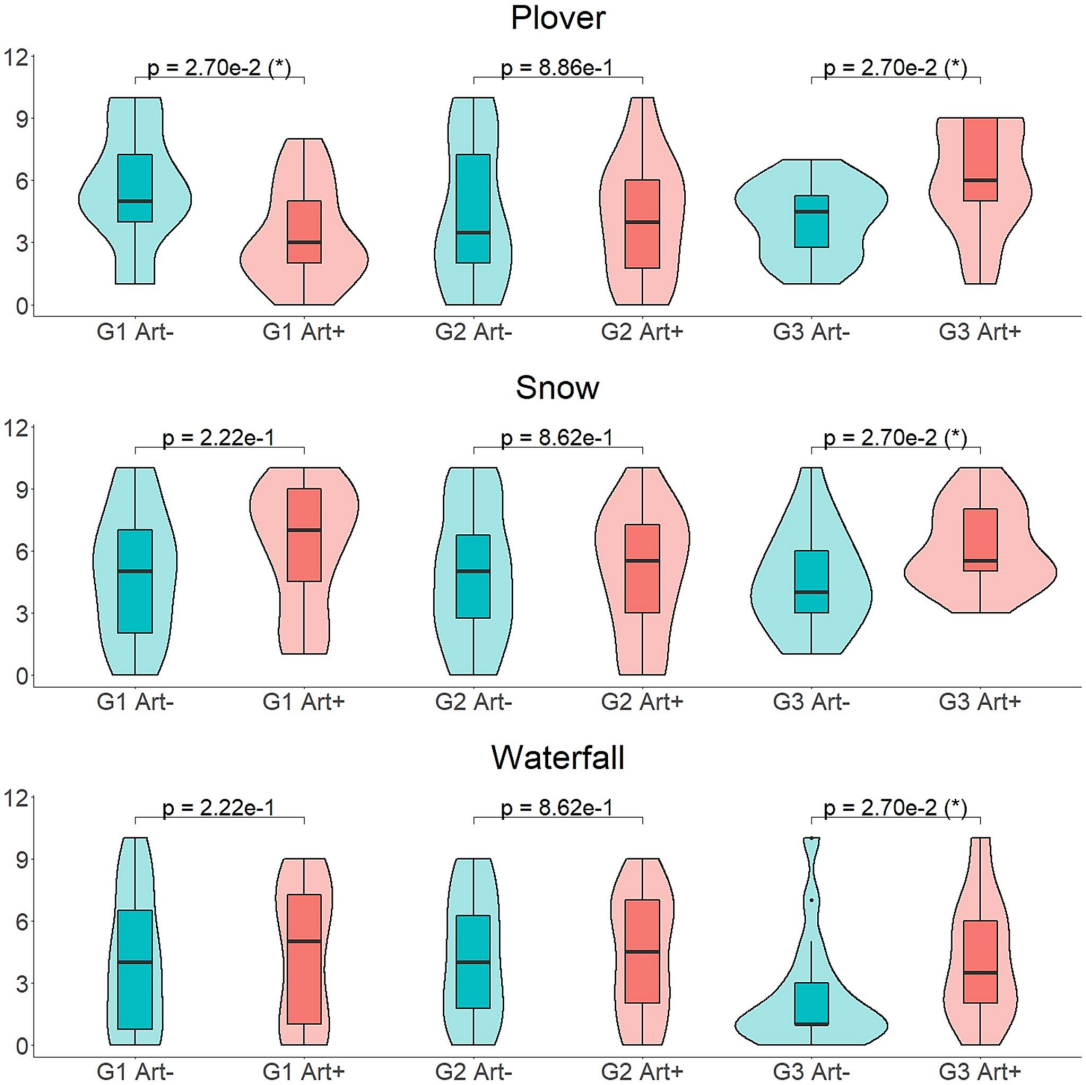
*Post-hoc* comparisons of the ratings of the descriptor *tension* in the different experimental groups, for the sounds of *plover, snow* and *waterfall*, using Wilcoxon Signed-Ranks tests to study the differences between the *art*+ and *art*− conditions. The *p*-values displayed have been corrected for multiple testing.

The ratings of the *tension* descriptor were significantly influenced by the triple interaction of the factors *art*, *group* and *sound* [*F*(32,912) = 2.241, *p* = 0.0001]. Thus, *post-hoc* analyses were performed analyzing the factor *art* in each group and sound, using Wilcoxon Signed-Ranks tests. A total of 51 tests were carried out, and after correcting the *p*-values for multiple comparisons, some significant differences were found between the ratings of the *art+* and *art−* versions of the sounds of the *plover*, the *snow* and the *waterfall* (Supplementary Table S7). Participants from the first group rated the sound of the *plover* in the *art+* condition –convolved with the IR recorded in front of the rock art panel of Kalbak-Tash I- as significantly less tension-evoking (*M* = 3.350, SD = 2.368) than in the *art−* (*M* = 5.650, SD = 2.834). On the contrary, in the third group, participants rated the sound of the *plover* in the *art+* condition -convolved with the IR recorded in front of the panel of Torgun I5- as more tension-evoking (*M* = 6.050, SD = 2.564) than in the *art−* (*M* = 4.150, SD = 1.725). Participants from the third group also rated the sounds of the *snow* and the *waterfall* as more tension-evoking in the *art+* condition (snow: *M* = 6.150, SD = 1.981; waterfall: *M* = 4.200, SD = 2.895) than in the *art−* (snow: *M* = 4.600, SD = 2.437; waterfall: *M* = 2.250, SD = 2.573; Figure 9).

### 3.2. Spectral analysis

**Figure 10:**
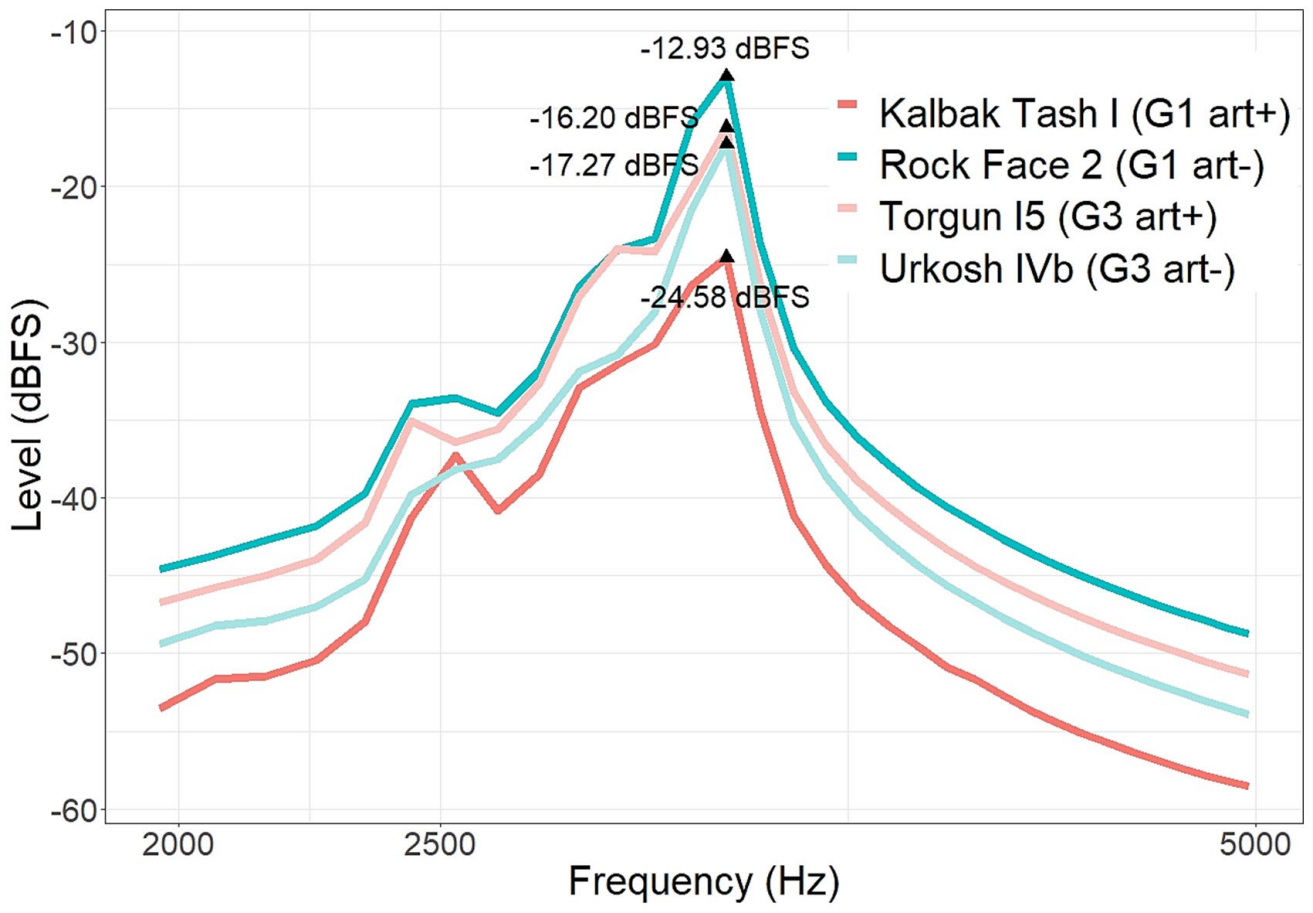
Spectral amplitude (dBFS) in the frequency range between 2000-5000 Hz of the sound of *plover* convolved with the four impulse responses forming the bundles 1 (dark colours) and 3 (light colours), listened by participants from groups 1 and 3 respectively, in *art*− (in front of a wall away from rock art panels, colour blue) and *art*+ (in front of a panel with rock art, colour pink) conditions.

Several studies have suggested a relationship between emotional valence elicited by sound and the energy distribution along the frequency spectrum (Scherer, 1986; Banse and Scherer, 1996; Waaramaa et al., 2010; Patel et al., 2011). Hence, we studied the spectrum of the sound of *plover* convolved with the impulse responses conforming the first and third bundles (Figure 10). We expected to observe an amplification of particular frequencies in some of its versions that could explain the effect of art acting in opposite direction in the first and third groups, as described in the previous section.

The peak frequency of the sound of *plover* was localized at 3188 Hz. The differences in amplitude at this frequency between the four versions of the sound -thus, convolved with the IRs- were studied. The *art+* version of the sound of plover that participants from the first group were presented with was 11.65 dB lower than the *art−* one at the peak frequency of 3,188 Hz. On the contrary, the *art+* version of the sound of plover that participants from the third group listened to was 1.08 dB louder than the *art−* one at the peak frequency.

The analysis of the ratings of *tension* attributed to the sounds of *snow* and *waterfall* did not provide more information than those obtained previously, in the analysis of the effect of the interaction between the factors *art* and *group*, which showed that participants from the third group experienced more *tension* when listening to the *art+* version of sounds. For this reason the frequency spectrum of the sounds of *snow* and *waterfall* was not studied.

## 4. Discussion

In this study, we have examined the perceptual and emotional reactions elicited by sounds from natural sources convolved with six impulse responses recorded in several rock art emplacements in Altai (Russia). We have observed the effect of the different acoustic prints in participants’ self-reported impressions using emotional and psychoacoustical scales, and studied if the perception of the acoustics of the site could have played any role in the selection for rock art production.

Participants from the third experimental group reported hearing the sounds as more present, closer and more tension-evoking when convolved with the impulse response recorded in front of the rock art panel of Torgun I5, compared to its *art−* version. Participants from the first group also reported their *art+* version of the sounds, convolved with the IR recorded in front of the rock art panel of Kalbak Tash I, as more tension-evoking than the corresponding *art−*. These results, at first sight, support the hypothesis that there are some perceivable differences between the *art+* and *art−* acoustics and that these differences are consistent across rock art sites, but it conflicts with the results from the *post-hoc* analysis of the tension evoked by each sound separately. While participants from the third experimental group reported feeling greater tension when listening to the sounds of *plover*, *snow* and *waterfall* convolved with the *art+* IR, participants from the first experimental group reported the opposite about the sound of *plover*: feeling less tension when listening to this sound convolved with the *art+* IR, compared to the *art−*.

The ratings of sounds convolved with the IR recorded in front of the rock art panel of Torgun I5, by participants from the third group, as closer and more present, can be related with reverberation parameters. Although the reverberation times of all the IRs used in the present study are considerably short, they are nevertheless longer in all the IRs recorded in front of rock panels without rock art (*art−*) than in the IRs recorded in front of rock art panels (*art+*), and the differences are above the Just Noticeable Difference (JND) for reverberation time (Bork, 2000; Martellotta et al., 2009). Since at constant sound level and distance, a longer reverberation entails a greater perceived distance from the source [Bronkhorst and Houtgast, 1999; Kolarik et al., 2016; Strutt Help. Reverberant Sound Level (From RT), 2020], this may explain why sounds convolved with the *art+* IR of the panel of Torgun I5, with shorter reverberation than its *art−*, are perceived as closer and more present. Reverberation could also explain the stronger feeling of tension elicited to participants from the first and third experimental groups by sounds convolved with the IRs recorded in front of the rock art panels of Kalbak-Tash I and Torgun I5. Reverberation is suspected to be inversely related to fear-like emotions (Mo et al., 2016) although this has not been strongly demonstrated. In the case of the IR recorded in Kalbak-Tash I, the stronger feeling of tension elicited can be also explained by the natural sound amplification produced in the rock art sites of the area, according to Díaz-Andreu et al. (2022a), since tension-like feelings are directly related with loudness (Granot and Eitan, 2011; Ministère de la Santé et des Services sociaux, 2016).

The results revealed by the analysis of the ratings of tension for each sound separately, in the case of the sounds of *snow* and *waterfall* seem consistent with this argument. However, we observed an odd behavior of the results of the sound of *plover*. While the third group reported feeling greater tension when listening to this sound convolved with the *art+* IR from the panel of Torgun I5, in line with the results described so far, the first group reported the opposite: feeling greater tension when listening to the *art−* version of the sound. If we observe the spectral density of this sound convolved with each IR from bundles 1 and 3 (see Figure 10), the amplitude at the peak frequency of 3,188 Hz is 1.08 dB greater in the *art+* version of the third bundle (convolved with the IR from the rock art panel of Torgun I5) compared to its corresponding *art−* IR. This difference is short but above the JND (Long, 2014). Nevertheless, in the case of the first bundle, the opposite happens: amplitude of the frequency peak at 3188 Hz is 11.65 dB greater in the *art−* version of the sound than in the corresponding *art+* (convolved with the IR from the rock art panel of Kalbak Tash I). As we stated previously, tension-like feelings are directly related with loudness (Granot and Eitan, 2011; Ministère de la Santé et des Services sociaux, 2016) and this relation is especially prominent when sounds are characterized by high frequencies (Ilie and Thompson, 2006; Juslin and Västfjäll, 2008; Granot and Eitan, 2011). Thus, in this case, the marked high-frequency peak amplification, especially in the first bundle of IRs, might be masking the slight difference in reverberation, and positioning as the main cue responsible for eliciting greater tension in the *art−* version of the sound of *plover*, in participants from the first experimental group. As we have confirmed, this one single result does not influence the general tendency observed when analyzing the effect of the factor art in the tension attributed to all the sounds by participants from the first experimental group.

As a first attempt to investigate the impact of archaeological sites acoustics on the selection of specific places for rock art paintings or carvings by past pre-state societies in modern listeners, and as previously alluded to in the introduction, the present study has a number of limitations. The first limitation is related to the IRs employed, as these were recorded a few thousand years after the actual moment of the rock art production, so that the landscapes might have suffered a series of modifications due to natural or human factors. Some sophisticated solutions to overcome this problem may involve 3D modeling to represent the sites of interest and to simulate the acoustic reflections to obtain virtual IRs. This process is feasible when dealing with ancient buildings such as odea and theaters (Manzetti, 2016; Weinzierl and Lepa, 2017), but much more difficult when dealing with landscapes.

A second limitation relates to the nature of the actual stimulus sets used in the present experiment. In order to reproduce a soundscape accurately, the sounds employed in the convolutions must be anechoic or quasi-anechoic (Farina and Ayalon, 2003). In the present study, anechoic recordings from natural Siberian sources, such as animals, were impossible to record, but a quasi-anechoic version may be obtained by the deconvolution of the recorded sound with the IR from the place where it was recorded (Gemba, 2014). This approach should also be considered in further research, particularly when natural sounds are employed. For these reasons, the sounds obtained from the convolutions might not be a completely accurate representation of the soundscapes in the moment when rock art was produced, and this must be taken into account when extracting conclusions regarding the possible reasons why these places, and not others, were decorated.

A final concern regards reductionism. The present experiment was set as an empirical investigation in the present of a far-reaching question: how past societies may have considered acoustic cues to place their sacred spaces, and spaces for social interaction, thousands of years ago. It is essential to pay close attention to the conclusions that can be obtained from such an approximation of the past in the present. Emotion, one of the main objects of study of this work, must be studied through the lens of culture. Certain acoustics might exalt certain emotions in modern-day listeners that might have been experienced by individuals in the past as well, but their cultural meanings, starting from what we consider *positive* and *negative* emotions, are probably very different. It is still not clear which emotions are liable to historical inquiry (Petri, 2013) and for this reason, we cannot fully understand yet the role of emotion in the process of selecting the spaces to produce the rock art. It has been previously mentioned that rock art is the mark we have in the present to determine the locales where social and ritual activities were most likely set (Díaz-Andreu and Mattioli, 2015), but the nature of such activities has radically evolved in its ways and its functions have also been transformed ever since. The concept of ritual itself has diverse meanings. While in modern western cultures ritual carries connotations of religion and sacredness, and it must be understood in the context of the modern knowledge, in the past myths and beliefs were the only way to understand the world and to organize a society (Koutrafouri, 2008). The problem of experimentation with modern-day participants is not only that the ritual-like situation is not recreated with all its variables, but also that it has never and never will be experienced by modern-day individuals in the same way it was at the moment when the rock art was produced. In summary, the hypothesis we set and the conclusions we reached can guide us in our study of the past, but will still relate to the present. Yet, to our understanding, this is one of the probably few approaches we have.

## 5. Conclusion

Based on the results obtained, our objectives have been partially met. On the one hand, an effect of the acoustic environment has been observed in some of the rock art sites studied over the ratings of presence, closeness and tension. On the other hand, however, only one effect has been found to be consistent across two of the three rock art sites: participants from the first and third experimental groups reported feeling greater tension when listening to the sounds convolved with the IRs from the recording positions in front of the rock art panels. We suggested the role of the reverberation times of the particular IRs used in this study as a potential explanation for this result. Thus, in future experiments, the range of psychoacoustical and emotional dimensions should be expanded, based on the differential acoustic features between the IRs from recording spots with and without rock art.

No commonalities were found between the three experimental groups. Therefore, further research is necessary in order to find more common features between rock art sites, from the acoustic, psychoacoustic and emotional point of view, to support the idea that acoustics was a factor to be taken into account when producing rock art. Finally, the inclusion of the factor of prehistoric period, or functionality of the rock art site might produce interesting results in the future.

## Data availability statement

The raw data supporting the conclusions of this article will be made available by the authors, without undue reservation.

## Ethics statement

The studies involving humans were approved by Bioethics Commission of the University of Barcelona. The studies were conducted in accordance with the local legislation and institutional requirements. The participants provided their written informed consent to participate in this study. Written informed consent was obtained from the individual(s) for the publication of any potentially identifiable images or data included in this article.

## Author contributions

SL-M, RA-T, and CE conceived the study and designed the experimental approach. SL-M acquired, analyzed, and interpreted the data. All authors contributed to the article and approved the submitted version.

## Funding

This work is part of the ERC Artsoundscapes project (grant agreement no. 787842) that has received funding from the European Research Council (ERC) under the European Union’s Horizon 2020 research and innovation program. PI: MD-A. CE was also supported by the Generalitat de Catalunya SGR2017-974, de María de Maeztu Center of Excellence (Institute of Neurosciences, University of Barcelona) CEX2021-001159-M, Ministry of Science and Innovation, and the ICREA Acadèmia Distinguished Professorship Award.

## Conflict of interest

The authors declare that the research was conducted in the absence of any commercial or financial relationships that could be construed as a potential conflict of interest.

## Publisher’s note

All claims expressed in this article are solely those of the authors and do not necessarily represent those of their affiliated organizations, or those of the publisher, the editors and the reviewers. Any product that may be evaluated in this article, or claim that may be made by its manufacturer, is not guaranteed or endorsed by the publisher.

## Footnotes

^1^Audacity (version 2.3.3) [Computer software]. USA. Retrieved from https://www.audacityteam.org/.

^2^ARTA Acoustic Software, by ARTA Labs: https://artalabs.hr/.

^3^Bidule, by Plogue INC, advanced modular audio software: https://www.plogue.com/products/bidule.html.

^4^X-MCFX Vst plugin, version 0.5.11 (2014). Mattias Kronlachner [Computer software]. Available online at: http://www.matthiaskronlachner.com/.
