# Supplementary material for: Acoustic perception and emotion evocation by rock art soundscapes of Altai (Russia)
Source: Front Psychol. 2023 Sep 19;14:1188567. doi: 10.3389/fpsyg.2023.1188567 (PMC10546042; doi:10.3389/fpsyg.2023.1188567)
Supplement: Supplementary file 1 [file Data_Sheet_1.docx]

Supplementary Material

Acoustic Perception and Emotion Evocation by Rock-art Soundscapes of Altai (Russia)

Samantha López-Mochales, Raquel Aparicio-Terrés, Margarita Díaz-Andreu, Carles Escera*

*** Correspondence:** Carles Escera: cescera@ub.edu

# Supplementary Table 1

**Supplementary Table 1.** The sounds employed in the study and its sources.

| Name | Description | Original file | Source | Available at | Licensing |
| --- | --- | --- | --- | --- | --- |
| BEAR | Brown bear | ‘Bear Sounds’ | YouTube, user Robert Hintz | https://www.youtube.com/watch?v=2U4EfY2odUc&t=63s | Used with permission. |
| CRANE | Siberian crane | ‘Grulla Siberiana’ | Xeno-Canto, user Oscar Campbell | https://xeno-canto.org/401979 | Creative Commons Attribution-Share Alike 4.0 International. |
| CRICKET | Roesel’s bush cricket | ‘Roesel’s bush-cricket' | Wikipedia, user K.vliet | https://en.wikipedia.org/wiki/File:Metrioptera_roeselii_-_sound.oga | Creative Commons Attribution-Share Alike 4.0 International. |
| EAGLE | Siberian Golden Eagle | ‘Golden Eagles Sounds and Pictures’ | YouTube, user ESL and Popular Culture | https://www.youtube.com/watch?v=66M4ZSjvGUQ&t=28s | Used with permission. |
| FIRE | Bonfire | ‘Open fire burning’ | BBC Database | https://sound-effects.bbcrewind.co.uk/search?q=07059050 | Copyright 2022 BBC. |
| IBEX | Alpine Ibex | ‘Three Rams Bleating – Sound Effect’ | YouTube, user MasterSoundEffects | https://www.youtube.com/watch?v=sRdCCjXkFRU | Used with permission. |
| LEOPARD | Snow leopard | ‘Snow Leopard Sounds’ | YouTube, user Alicat33 | https://www.youtube.com/watch?v=Y-LTMyg5WAw | Used with permission. |
| LYNX | Eurasian lynx | ‘Lynx sounds / Lynx scream at night / Lynx voice / Will lynx call and sound’ | YouTube, user The Animal Sounds | https://www.youtube.com/watch?v=R7oi579cUXA&t=46s | Used with permission. |
| PLOVER | Siberian plover | ‘Chorlito dorado siberiano’ | Xeno-Canto, user Jens Kirkeby | https://xeno-canto.org/622920 | Creative Commons Attribution-Share Alike 4.0 International. |
| RAIN | Light rain | ‘Tropical Storm, rain and heavy thunder recorded from aprtment balcony’ | BBC Database | https://sound-effects.bbcrewind.co.uk/search?q=07070202 | Copyright 2022 BBC. |
| RIVER | Small river flow | ‘Small river running over rocks – 1974 (1W6, reprocessed)’ | BBC Database | https://sound-effects.bbcrewind.co.uk/search?q=07060073 | Copyright 2022 BBC. |
| SHEEP | Argali sheep | ‘Bighorn sheep’ | National Parks Service (US) | https://www.nps.gov/subjects/sound/sounds-bighorn-ram.htm | Public Domain (Copyright Free). |
| SNOW | Snowstorm | ‘Snowstorm-heavy snow in swirling wind’ | BBC Database | https://sound-effects.bbcrewind.co.uk/search?q=07061030 | Copyright 2022 BBC. |
| THUNDER | Thunderstorm | ‘Severe thunderstorm with light rain’ | BBC Database | https://sound-effects.bbcrewind.co.uk/search?q=07043401 | Copyright 2022 BBC. |
| WATERFALL | Waterfall | ‘Weir in full flood (1W8, reprocessed)’ | BBC Database | https://sound-effects.bbcrewind.co.uk/search?q=07064029 | Copyright 2022 BBC. |
| WIND | Wind blowing | ‘Mixed deciduous woodland atmosphere on a windy day with occasional flying insects’ | BBC Database | https://sound-effects.bbcrewind.co.uk/search?q=07044012 | Copyright 2022 BBC. |
| WOLVES | Pack of 5 grey wolves | ‘wolfs.wav’ | Freesound, user ondrosik | https://freesound.org/people/ondrosik/sounds/143568/ | Creative Commons Attribution-Share Alike 4.0 International. |

# Supplementary Table 2

**Supplementary Table 2.** Detailed information of the sounds employed in the practice trials and its sources.

| Name | Description | Original file | Source | Available at | Licensing |
| --- | --- | --- | --- | --- | --- |
| RANDOM_BIRD | Dunnock | ‘Dunnock (Prunella Modularis) - song with other birds incl. Gulls in background’ | BBC Database | https://sound-effects.bbcrewind.co.uk/search?q=NHU10717583 | Copyright 2022 BBC. |
| RANDOM_MONKEY | Baboon | ‘Gelada Baboon (Theropithecus Gelada) - medium close-up excitable calls’ | BBC Database | https://sound-effects.bbcrewind.co.uk/search?q=NHU9719732 | Copyright 2022 BBC. |
| RANDOM_RIVER | River | ‘River Atmosphere – medium close-up fast flowing river; gulls calling in background’ | BBC Database | https://sound-effects.bbcrewind.co.uk/search?q=NHU9707707 | Copyright 2022 BBC. |
| RANDOM_FOREST | Forest atmosphere | ‘Forest Atmosphere – neutral atmosphere w/ birds; distant rumble’ | BBC Database | https://sound-effects.bbcrewind.co.uk/search?q=NHU9710303 | Copyright 2022 BBC. |
| RANDOM_IMPALA | Impala | ‘Impala (Aepyceros Melampus) - close-up grunting; sound of hooves’ | BBC Database | https://sound-effects.bbcrewind.co.uk/search?q=NHU9753298 | Copyright 2022 BBC. |

# Supplementary Table 3

**Supplementary Table 3.** Detailed information of the sounds employed in the pilot and its sources.

| Name | Description | Original file | Source | Available at | Licensing |
| --- | --- | --- | --- | --- | --- |
| BIRDS | Robin singing | ‘Animals And Birds - Robin singing.’ | BBC Database | https://sound-effects.bbcrewind.co.uk/search?q=07042130 | Copyright 2022 BBC. |
| CRICKET | Cricket sound | ‘Cricket Sp - close-up stridulating in hole in mud by ditch.’ | BBC Database | https://sound-effects.bbcrewind.co.uk/search?q=NHU05093110 | Copyright 2022 BBC. |
| FOREST | Ambient forest sound | ‘Beech Forest Atmosphere - Southern beech forest. Sedge wren, thorn-tailed rayaditos and rufous-collared sparrow, almost no wind.’ | BBC Database | https://sound-effects.bbcrewind.co.uk/search?q=NHU05074159 | Copyright 2022 BBC. |
| PIGS | Pigs grunting and eating | ‘Pigs - Exterior - peasant farm, pig in pigpen grunting and eating in medium close-up (rec. in Romania).’ | BBC Database | https://sound-effects.bbcrewind.co.uk/search?q=07068058 | Copyright 2022 BBC. |
| WATER | Trickling water sound | ‘Water - close-up sound of trickling water in stream. Very distant bird calls. Distant sounds of flowing river.’ | BBC Database | https://sound-effects.bbcrewind.co.uk/search?q=NHU05013029 | Copyright 2022 BBC. |

# Supplementary Table 4

**Supplementary Table 4.** Results of the questionnaires’ scorings’ effects on each descriptor’s rating, using linear regression.

| **Present~sSTAI** | | | | | | |
| --- | --- | --- | --- | --- | --- | --- |
|  |  | Estimate | Std. | Error | Corrected p-value | R^2^ |
|  | Intercept | 7.699 | 0.089 | 86.986 | 2.000E-16 | 0.033 |
|  | sSTAI | -0.049 | 0.006 | -8.352 | 2.000E-16 |  |
| **Present~tSTAI** | | | | | | |
|  |  | Estimate | Std. | Error | Corrected p-value | R^2^ |
|  | Intercept | 7.520 | 0.103 | 72.876 | 2.000E-16 | 0.011 |
|  | tSTAI | -0.022 | 0.005 | -4.845 | 1.360E-06 |  |
| **Present~religiosity** | | | | | | |
|  |  | Estimate | Std. | Error | Corrected p-value | R^2^ |
|  | Intercept | 6.943 | 0.075 | 92.480 | 2.000E-16 | 0.002 |
|  | religiosity | 0.029 | 0.012 | 2.380 | 1.740E-02 |  |
| **Spatious~sSTAI** | | | | | | |
|  |  | Estimate | Std. | Error | Corrected p-value | R^2^ |
|  | Intercept | 6.570 | 0.105 | 62.510 | 2.000E-16 | 0.004 |
|  | sSTAI | -0.022 | 0.007 | -3.097 | 1.980E-03 |  |
| **Spatious~tSTAI** | | | | | | |
|  |  | Estimate | Std. | Error | Corrected p-value | R^2^ |
|  | Intercept | 6.558 | 0.121 | 54.080 | 2.000E-16 | 0.002 |
|  | tSTAI | -0.013 | 0.005 | -2.449 | 1.440E-02 |  |
| **Spatious~religiosity** | | | | | | |
|  |  | Estimate | Std. | Error | Corrected p-value | R^2^ |
|  | Intercept | 5.943 | 0.087 | 68.020 | 2.000E-16 | 0.013 |
|  | religiosity | 0.075 | 0.014 | 5.325 | 1.120E-07 |  |
| **Enveloping~sSTAI** | | | | | | |
|  | | Estimate | Std. | Error | Corrected p-value | R^2^ |
|  | Intercept | 7.103 | 0.103 | 68.708 | 2.000E-16 | 0.023 |
|  | sSTAI | -0.048 | 0.007 | -7.013 | 3.170E-12 |  |
| **Enveloping~tSTAI** | | | | | | |
|  |  | Estimate | Std. | Error | Corrected p-value | R^2^ |
|  | Intercept | 7.011 | 0.120 | 58.471 | 2.000E-16 | 0.011 |
|  | tSTAI | -0.026 | 0.005 | -4.888 | 1.100E-06 |  |
| **Enveloping~religiosity** | | | | | | |
|  |  | Estimate | Std. | Error | Corrected p-value | R^2^ |
|  | Intercept | 6.084 | 0.087 | 70.296 | 2.000E-16 | 0.018 |
|  | religiosity | 0.087 | 0.014 | 6.247 | 5.090E-10 |  |
| **Deep~sSTAI** | | | | | | |
|  |  | Estimate | Std. | Error | Corrected p-value | R^2^ |
|  | Intercept | 5.659 | 0.115 | 49.414 | 2.000E-16 | 0.005 |
|  | sSTAI | -0.027 | 0.008 | -3.484 | 5.050E-04 |  |
| **Deep~tSTAI** | | | | | | |
|  |  | Estimate | Std. | Error | Corrected p-value | R^2^ |
|  | Intercept | 5.494 | 0.132 | 41.510 | 2.000E-16 | 0.001 |
|  | tSTAI | -0.008 | 0.006 | -1.456 | 1.460E-01 |  |
| **Deep~religiosity** | | | | | | |
|  |  | Estimate | Std. | Error | Corrected p-value | R^2^ |
|  | Intercept | 5.020 | 0.096 | 52.550 | 2.000E-16 | 0.008 |
|  | religiosity | 0.064 | 0.015 | 4.200 | 2.790E-05 |  |
| **Close~sSTAI** | | | | | | |
|  |  | Estimate | Std. | Error | Corrected p-value | R^2^ |
|  | Intercept | 7.397 | 0.091 | 80.884 | 2.000E-16 | 0.034 |
|  | sSTAI | -0.052 | 0.006 | -8.569 | 2.000E-16 |  |
| **Close~tSTAI** | | | | | | |
|  |  | Estimate | Std. | Error | Corrected p-value | R^2^ |
|  | Intercept | 7.208 | 0.107 | 67.570 | 2.000E-16 | 0.012 |
|  | tSTAI | -0.023 | 0.005 | -4.979 | 6.930E-07 |  |
| **Close~religiosity** | | | | | | |
|  |  | Estimate | Std. | Error | Corrected p-value | R^2^ |
|  | Intercept | 6.878 | 0.078 | 88.582 | 2.000E-16 | 0.002 |
|  | religiosity | -0.029 | 0.012 | -2.343 | 1.920E-02 |  |
| **Alert~sSTAI** | | | | | | |
|  |  | Estimate | Std. | Error | Corrected p-value | R^2^ |
|  | Intercept | 5.073 | 0.129 | 39.208 | 2.000E-16 | 0.000 |
|  | sSTAI | -0.003 | 0.009 | -0.357 | 7.210E-01 |  |
| **Alert~tSTAI** | | | | | | |
|  |  | Estimate | Std. | Error | Corrected p-value | R^2^ |
|  | Intercept | 4.707 | 0.149 | 31.604 | 2.000E-16 | 0.003 |
|  | tSTAI | 0.016 | 0.007 | 2.489 | 1.290E-02 |  |
| **Alert~religiosity** | | | | | | |
|  |  | Estimate | Std. | Error | Corrected p-value | R^2^ |
|  | Intercept | 4.901 | 0.108 | 45.384 | 2.000E-16 | 0.001 |
|  | religiosity | 0.028 | 0.017 | 1.621 | 1.050E-01 |  |
| **Tension~sSTAI** | | | | | | |
|  |  | Estimate | Std. | Error | Corrected p-value | R^2^ |
|  | Intercept | 5.119 | 0.133 | 38.522 | 2.000E-16 | 0.000 |
|  | sSTAI | -0.003 | 0.009 | -0.329 | 7.420E-01 |  |
| **Tension~tSTAI** | | | | | | |
|  |  | Estimate | Std. | Error | Corrected p-value | R^2^ |
|  | Intercept | 4.769 | 0.153 | 31.173 | 2.000E-16 | 0.002 |
|  | tSTAI | 0.016 | 0.007 | 2.318 | 2.060E-02 |  |
| **Tension~religiosity** | | | | | | |
|  |  | Estimate | Std. | Error | Corrected p-value | R^2^ |
|  | Intercept | 5.043 | 0.111 | 45.446 | 2.000E-16 | 0.000 |
|  | religiosity | 0.008 | 0.018 | 0.459 | 6.470E-01 |  |
| **Peace~sSTAI** | | | | | | |
|  |  | Estimate | Std. | Error | Corrected p-value | R^2^ |
|  | Intercept | 4.320 | 0.130 | 33.150 | 2.000E-16 | 0.008 |
|  | sSTAI | -0.037 | 0.009 | -4.280 | 1.960E-05 |  |
| **Peace~tSTAI** | | | | | | |
|  |  | Estimate | Std. | Error | Corrected p-value | R^2^ |
|  | Intercept | 4.047 | 0.151 | 26.833 | 2.000E-16 | 0.001 |
|  | tSTAI | -0.010 | 0.007 | -1.466 | 1.430E-01 |  |
| **Peace~religiosity** | | | | | | |
|  |  | Estimate | Std. | Error | Corrected p-value | R^2^ |
|  | Intercept | 3.506 | 0.109 | 32.207 | 2.000E-16 | 0.008 |
|  | religiosity | 0.073 | 0.017 | 4.193 | 2.870E-05 |  |
| **Calm~sSTAI** | | | | | | |
|  |  | Estimate | Std. | Error | Corrected p-value | R^2^ |
|  | Intercept | 4.378 | 0.129 | 33.924 | 2.000E-16 | 0.009 |
|  | sSTAI | -0.037 | 0.009 | -4.307 | 1.730E-05 |  |
| **Calm~tSTAI** | | | | | | |
|  |  | Estimate | Std. | Error | Corrected p-value | R^2^ |
|  | Intercept | 4.197 | 0.149 | 28.112 | 2.000E-16 | 0.002 |
|  | tSTAI | -0.014 | 0.007 | -2.167 | 3.030E-02 |  |
| **Calm~religiosity** | | | | | | |
|  |  | Estimate | Std. | Error | Corrected p-value | R^2^ |
|  | Intercept | 3.599 | 0.108 | 33.358 | 2.000E-16 | 0.007 |
|  | religiosity | 0.066 | 0.017 | 3.819 | 1.380E-04 |  |
| **Pleasure~sSTAI** | | | | | | |
|  |  | Estimate | Std. | Error | Corrected p-value | R^2^ |
|  | Intercept | 4.201 | 0.134 | 31.405 | 2.000E-16 | 0.003 |
|  | sSTAI | -0.025 | 0.009 | -2.838 | 4.580E-03 |  |
| **Pleasure~tSTAI** | | | | | | |
|  |  | Estimate | Std. | Error | Corrected p-value | R^2^ |
|  | Intercept | 3.700 | 0.154 | 23.953 | 2.000E-16 | 0.000 |
|  | tSTAI | 0.009 | 0.007 | 1.342 | 1.800E-01 |  |
| **Pleasure~religiosity** | | | | | | |
|  |  | Estimate | Std. | Error | Corrected p-value | R^2^ |
|  | Intercept | 3.600 | 0.112 | 32.243 | 2.000E-16 | 0.005 |
|  | religiosity | 0.060 | 0.018 | 3.339 | 8.570E-04 |  |

# Supplementary Table 5

**Supplementary Table 5.** Results of the mixed ANOVA analyses applied on the data from the descriptors’ ratings in response to the sounds.

| **Descriptor: PRESENT** | | | | | | | |
| --- | --- | --- | --- | --- | --- | --- | --- |
| Factor | DFn | DFd | SSn | SSd | F | Corrected p-value | η^2^ |
| Intercept | 1 | 57 | 102226.706 | 3830.907 | 1521.029 | 2.11E-42 | 0.917 |
| group | 2 | 57 | 44.004 | 3830.907 | 0.327 | 9.38E-01 | 0.005 |
| art | 1 | 57 | 33.393 | 392.860 | 4.845 | 4.54E-02* | 0.004 |
| sound | 16 | 912 | 274.219 | 3031.218 | 5.156 | 1.98E-10*** | 0.029 |
| group:art | 2 | 57 | 88.424 | 392.860 | 6.415 | 1.54E-02* | 0.009 |
| group:sound | 32 | 912 | 102.446 | 3031.218 | 0.963 | 8.00E-01 | 0.011 |
| art:sound | 16 | 912 | 17.932 | 2042.265 | 0.500 | 9.68E-01 | 0.002 |
| gr.:art:sound | 32 | 912 | 106.626 | 2042.265 | 1.488 | 1.99E-01 | 0.011 |
| **Descriptor: SPATIOUS** | | | | | | | |
| Factor | DFn | DFd | SSn | SSd | F | Corrected p-value | η^2^ |
| Intercept | 1 | 57 | 80879.424 | 2199.599 | 2095.895 | 1.20E-45 | 0.878 |
| group | 2 | 57 | 22.301 | 2199.599 | 0.289 | 9.38E-01 | 0.002 |
| art | 1 | 57 | 6.040 | 456.557 | 0.754 | 3.89E-01 | 0.001 |
| sound | 16 | 912 | 2040.434 | 5989.127 | 19.419 | 7.67E-48*** | 0.154 |
| group:art | 2 | 57 | 2.491 | 456.557 | 0.156 | 8.56E-01 | 0.000 |
| group:sound | 32 | 912 | 258.616 | 5989.127 | 1.231 | 5.40E-01 | 0.023 |
| art:sound | 16 | 912 | 20.152 | 2549.468 | 0.451 | 9.68E-01 | 0.002 |
| gr.:art:sound | 32 | 912 | 102.792 | 2549.468 | 1.149 | 3.74E-01 | 0.009 |
| **Descriptor: ENVELOPING** | | | | | | | |
| Factor | DFn | DFd | SSn | SSd | F | Corrected p-value | η^2^ |
| Intercept | 1 | 57 | 86047.059 | 2899.002 | 1691.852 | 2.25E-43 | 0.879 |
| group | 2 | 57 | 30.145 | 2899.002 | 0.296 | 9.38E-01 | 0.003 |
| art | 1 | 57 | 28.471 | 750.507 | 2.162 | 1.84E-01 | 0.002 |
| sound | 16 | 912 | 1133.549 | 5862.224 | 11.022 | 3.00E-26*** | 0.087 |
| group:art | 2 | 57 | 19.816 | 750.507 | 0.752 | 5.29E-01 | 0.002 |
| group:sound | 32 | 912 | 249.522 | 5862.224 | 1.213 | 5.40E-01 | 0.021 |
| art:sound | 16 | 912 | 69.804 | 2322.418 | 1.713 | 2.60E-01 | 0.006 |
| gr.:art:sound | 32 | 912 | 94.484 | 2322.418 | 1.159 | 3.74E-01 | 0.008 |
| **Descriptor: DEEP** | | | | | | | |
| Factor | DFn | DFd | SSn | SSd | F | Corrected p-value | η^2^ |
| Intercept | 1 | 57 | 57834.825 | 2860.159 | 1152.588 | 3.32E-39 | 0.819 |
| group | 2 | 57 | 82.545 | 2860.159 | 0.823 | 9.38E-01 | 0.006 |
| art | 1 | 57 | 13.184 | 586.647 | 1.281 | 2.91E-01 | 0.001 |
| sound | 16 | 912 | 2958.458 | 6902.341 | 24.431 | 7.47E-60*** | 0.188 |
| group:art | 2 | 57 | 27.345 | 586.647 | 1.328 | 3.90E-01 | 0.002 |
| group:sound | 32 | 912 | 214.672 | 6902.341 | 0.886 | 8.00E-01 | 0.016 |
| art:sound | 16 | 912 | 44.199 | 2451.553 | 1.028 | 6.06E-01 | 0.003 |
| gr.:art:sound | 32 | 912 | 86.072 | 2451.553 | 1.001 | 5.20E-01 | 0.007 |
| **Descriptor: CLOSE** | | | | | | | |
| Factor | DFn | DFd | SSn | SSd | F | Corrected p-value | η^2^ |
| Intercept | 1 | 57 | 92664.216 | 3308.487 | 1596.458 | 7.43E-43 | 0.908 |
| group | 2 | 57 | 12.503 | 3308.487 | 0.108 | 9.47E-01 | 0.001 |
| art | 1 | 57 | 78.040 | 280.263 | 15.872 | 9.75E-04** | 0.008 |
| sound | 16 | 912 | 759.859 | 3638.938 | 11.902 | 1.49E-28*** | 0.075 |
| group:art | 2 | 57 | 113.726 | 280.263 | 11.565 | 6.09E-04** | 0.012 |
| group:sound | 32 | 912 | 193.497 | 3638.938 | 1.515 | 3.42E-01 | 0.020 |
| art:sound | 16 | 912 | 41.935 | 2123.162 | 1.126 | 6.06E-01 | 0.004 |
| gr.:art:sound | 32 | 912 | 106.374 | 2123.162 | 1.428 | 1.99E-01 | 0.011 |
| **Descriptor: ALERT** | | | | | | | |
| Factor | DFn | DFd | SSn | SSd | F | Corrected p-value | η^2^ |
| Intercept | 1 | 57 | 51702.402 | 3536.053 | 833.426 | 1.47E-35 | 0.803 |
| group | 2 | 57 | 143.545 | 3536.053 | 1.157 | 9.38E-01 | 0.011 |
| art | 1 | 57 | 36.267 | 254.247 | 8.131 | 1.01E-02* | 0.003 |
| sound | 16 | 912 | 7330.281 | 6773.397 | 61.686 | 1.69E-132*** | 0.366 |
| group:art | 2 | 57 | 35.839 | 254.247 | 4.017 | 5.83E-02 | 0.003 |
| group:sound | 32 | 912 | 235.322 | 6773.397 | 0.990 | 8.00E-01 | 0.018 |
| art:sound | 16 | 912 | 38.417 | 2111.603 | 1.037 | 6.06E-01 | 0.003 |
| gr.:art:sound | 32 | 912 | 94.627 | 2111.603 | 1.277 | 2.82E-01 | 0.007 |
| **Descriptor: TENSION** | | | | | | | |
| Factor | DFn | DFd | SSn | SSd | F | Corrected p-value | η^2^ |
| Intercept | 1 | 57 | 52683.671 | 2746.763 | 1093.276 | 1.16E-38 | 0.806 |
| group | 2 | 57 | 47.301 | 2746.763 | 0.491 | 9.38E-01 | 0.004 |
| art | 1 | 57 | 136.142 | 278.381 | 27.876 | 2.10E-05*** | 0.011 |
| sound | 16 | 912 | 8217.621 | 7437.112 | 62.982 | 1.93E-134*** | 0.393 |
| group:art | 2 | 57 | 55.213 | 278.381 | 5.653 | 1.92E-02* | 0.004 |
| group:sound | 32 | 912 | 311.032 | 7437.112 | 1.192 | 5.40E-01 | 0.024 |
| art:sound | 16 | 912 | 57.950 | 2248.994 | 1.469 | 2.60E-01 | 0.005 |
| gr.:art:sound | 32 | 912 | 176.821 | 2248.994 | 2.241 | 1.13E-03** | 0.014 |
| **Descriptor: PEACE** | | | | | | | |
| Factor | DFn | DFd | SSn | SSd | F | Corrected p-value | η^2^ |
| Intercept | 1 | 57 | 30276.412 | 3563.154 | 484.334 | 1.53E-29 | 0.696 |
| group | 2 | 57 | 6.757 | 3563.154 | 0.054 | 9.47E-01 | 0.001 |
| art | 1 | 57 | 70.412 | 308.119 | 13.026 | 2.00E-03** | 0.005 |
| sound | 16 | 912 | 7372.029 | 7219.971 | 58.200 | 8.35E-127*** | 0.358 |
| group:art | 2 | 57 | 30.027 | 308.119 | 2.777 | 1.18E-01 | 0.002 |
| group:sound | 32 | 912 | 206.176 | 7219.971 | 0.814 | 8.00E-01 | 0.015 |
| art:sound | 16 | 912 | 56.663 | 2138.406 | 1.510 | 2.60E-01 | 0.004 |
| gr.:art:sound | 32 | 912 | 100.873 | 2138.406 | 1.344 | 2.44E-01 | 0.008 |
| **Descriptor: CALM** | | | | | | | |
| Factor | DFn | DFd | SSn | SSd | F | Corrected p-value | η^2^ |
| Intercept | 1 | 57 | 31215.882 | 2387.012 | 745.411 | 2.53E-34 | 0.721 |
| group | 2 | 57 | 31.812 | 2387.012 | 0.380 | 9.38E-01 | 0.003 |
| art | 1 | 57 | 61.429 | 278.994 | 12.550 | 2.00E-03** | 0.005 |
| sound | 16 | 912 | 8156.501 | 7332.188 | 63.408 | 7.82E-135*** | 0.403 |
| group:art | 2 | 57 | 31.812 | 278.994 | 3.250 | 9.22E-02 | 0.003 |
| group:sound | 32 | 912 | 201.605 | 7332.188 | 0.784 | 8.00E-01 | 0.016 |
| art:sound | 16 | 912 | 58.754 | 2061.206 | 1.625 | 2.60E-01 | 0.005 |
| gr.:art:sound | 32 | 912 | 68.805 | 2061.206 | 0.951 | 5.46E-01 | 0.006 |
| **Descriptor: PLEASURE** | | | | | | | |
| Factor | DFn | DFd | SSn | SSd | F | Corrected p-value | η^2^ |
| Intercept | 1 | 57 | 30756.000 | 3160.143 | 554.751 | 5.17E-31 | 0.693 |
| group | 2 | 57 | 64.827 | 3160.143 | 0.585 | 9.38E-01 | 0.005 |
| art | 1 | 57 | 48.024 | 260.637 | 10.503 | 3.98E-03** | 0.004 |
| sound | 16 | 912 | 8023.791 | 8249.482 | 55.441 | 3.58E-122*** | 0.371 |
| group:art | 2 | 57 | 7.604 | 260.637 | 0.831 | 5.29E-01 | 0.001 |
| group:sound | 32 | 912 | 248.256 | 8249.482 | 0.858 | 8.00E-01 | 0.018 |
| art:sound | 16 | 912 | 20.934 | 1940.088 | 0.615 | 9.68E-01 | 0.002 |
| gr.:art:sound | 32 | 912 | 73.213 | 1940.088 | 1.075 | 4.45E-01 | 0.005 |

# Supplementary Table 6

**Supplementary Table 6.** Results of the post-hoc analysis of the ratings of the descriptors *present*, *close* and *tension* in the different experimental groups, using Wilcoxon Signed-Ranks tests to study the differences between the *art+* and *art-* conditions.

| **Descriptor: PRESENT** | | | |
| --- | --- | --- | --- |
|  |  | V | Corrected p-value |
|  | Group 1 | 14520 | 6.77E-01 |
|  | Group 2 | 14276 | 8.67E-01 |
|  | Group 3 | 8366 | 7.65E-13*** |
| **Descriptor: CLOSE** | | | |
|  |  | V | Corrected p-value |
|  | Group 1 | 14842 | 9.25E-01 |
|  | Group 2 | 15115 | 7.07E-01 |
|  | Group 3 | 7822.5 | 6.60E-16*** |
| **Descriptor: TENSION** | | | |
|  |  | V | Corrected p-value |
|  | Group 1 | 10810 | 8.85E-03* |
|  | Group 2 | 13590 | 9.85E-02 |
|  | Group 3 | 5219.5 | 6.60E-16*** |

# Supplementary Table 7

**Supplementary Table 7.** Results of the post-hoc analysis of the ratings of the descriptor *tension* in the different experimental groups, for each one of the sounds, using Wilcoxon Signed-Ranks tests to study the differences between the *art+* and *art-* conditions.

| **Group** | **Sound** | **V** | **Corrected p-value** |
| --- | --- | --- | --- |
| **Group 1** | bear | 31 | 5.26E-01 |
|  | crane | 35.5 | 5.26E-01 |
|  | cricket | 4 | 1.79E-01 |
|  | eagle | 82 | 7.02E-01 |
|  | fire | 33.5 | 8.62E-01 |
|  | ibex | 48 | 7.11E-01 |
|  | leopard | 35 | 7.02E-01 |
|  | lynx | 22.5 | 5.74E-01 |
|  | plover | 165 | 2.70E-02* |
|  | rain | 9 | 1.79E-01 |
|  | river | 16 | 4.92E-01 |
|  | sheep | 61.5 | 8.73E-01 |
|  | snow | 52.5 | 2.22E-01 |
|  | thunder | 37 | 1.79E-01 |
|  | waterfall | 36 | 7.19E-01 |
|  | wind | 41.5 | 1.79E-01 |
|  | wolves | 13.5 | 1.34E-01 |
| **Group 2** | bear | 20.5 | 8.86E-01 |
|  | crane | 50 | 4.07E-01 |
|  | cricket | 113 | 2.22E-01 |
|  | eagle | 25 | 1.75E-01 |
|  | fire | 75.5 | 8.62E-01 |
|  | ibex | 22.5 | 8.35E-01 |
|  | leopard | 81.5 | 8.86E-01 |
|  | lynx | 53 | 2.22E-01 |
|  | plover | 90 | 8.86E-01 |
|  | rain | 48 | 8.86E-01 |
|  | river | 12 | 6.77E-01 |
|  | sheep | 36 | 5.26E-01 |
|  | snow | 46.5 | 8.62E-01 |
|  | thunder | 58.5 | 8.62E-01 |
|  | waterfall | 62.5 | 8.76E-01 |
|  | wind | 28.5 | 3.05E-01 |
|  | wolves | 62.5 | 8.76E-01 |
| **Group 3** | bear | 12 | 1.59E-01 |
|  | crane | 22.5 | 4.07E-01 |
|  | cricket | 33 | 3.01E-01 |
|  | eagle | 20.5 | 5.81E-02 |
|  | fire | 31 | 7.39E-01 |
|  | ibex | 55.5 | 5.37E-01 |
|  | leopard | 4.5 | 5.81E-02 |
|  | lynx | 15 | 6.01E-02 |
|  | plover | 4 | 2.70E-02* |
|  | rain | 8 | 1.74E-01 |
|  | river | 24 | 1.64E-01 |
|  | sheep | 35.5 | 8.76E-01 |
|  | snow | 7 | 2.70E-02* |
|  | thunder | 13.5 | 5.81E-02 |
|  | waterfall | 8 | 3.92E-02* |
|  | wind | 21 | 3.40E-01 |
|  | wolves | 23 | 1.12E-01 |
